# Supplementary material for: Feasibility and preliminary effects of a mindfulness-based physical exercise (MBPE) program for community-dwelling older people with sarcopenia: A protocol for a parallel, two-armed pilot randomised controlled trial
Source: PLoS One. 2024 Apr 18;19(4):e0302235. doi: 10.1371/journal.pone.0302235 (PMC11025830; doi:10.1371/journal.pone.0302235)
Supplement: S2 Appendix — (DOCX) [file pone.0302235.s002.docx]

**Informed consent form**

**Mindfulness-based physical exercise (MBPE) programme for community-dwelling older people with sarcopenia: protocol for a pilot randomised controlled trial**

You are invited to participate in a study conducted by Ms Mengli LI, a PhD student of The Hong Kong Polytechnic University, School of Nursing. Ms Mengli LI is under the supervision of Dr. Patrick KOR, an assistant professor and Dr. Justina LIU, an associate professor of The Hong Kong Polytechnic University, School of Nursing.

**Background**

Sarcopenia is a geriatric syndrome characterized by low muscle mass, muscle strength and physical function. It increases the risks of various health adverse events, such as falling, fracture, hospitalization. Exercise is the primary way to treat sarcopenia. While the older people with sarcopenia usually are unwilling to engage in any exercise and easier to have negative feeling when exercising. Mindfulness is able to help to better notice and control their feelings, improving the experience of exercise and thus the motivation to exercise.

**Objective**

The objective of this study is to evaluate the effect of a mindfulness-based physical activity intervention on physical and psychological health in older people with sarcopenia in the community.

**Study content**

If you meet the eligible criteria and agree to participate in this study, you will be randomized into control group or intervention group. For the intervention group, you will receive the intervention containing mindfulness and physical exercise, 2 sessions one week, about 60 minutes one session. The intervention will be conducted by a qualified mindfulness instructor and a sport coach. For the control group, you will receive health education for 12 weeks and if you are interested in the mindfulness-based physical exercise intervention, you can get all the intervention materials after the completion of this program.

**Data collection**

Participants of both groups will be invited to have some assessments including body composition, physical function, muscle strength and fill out some questionnaires before, and immediately after the intervention. This will be conducted at the community health care canter and will take about 30 minutes.

**Risk or Discomfort**

This study has a very little chance to cause you any adverse effects. The biggest possibility is minor muscle sore at the beginning of exercise, which is a normal body reaction and will fade away after some rest. However, if muscle soreness or other discomfort does occur, a professional rehabilitation therapist will be referred for further assessment.

**Privacy**

All the data will be safely kept by the researcher. All the personal information will be hidden whenever the data is used.

**Right**

You have the right to not participate in this study and withdraw at any time. Your medical rights won’t be affected.

If you would like to know more about this study or have any questions, please contact Ms Mengli LI at 18351075005.

Thank you for your interest in engaging this study.

I (name) agree to participate in this study conducted by Ms Mengli LI, supervised by Dr Patrick KOR and Dr. Justina LIU, The Hong Kong Polytechnic University, School of Nursing.

I understand that information obtained from this research may be used in future research and published. However, my right to privacy will be retained, i.e., my personal information will not be released.

The procedure in the attached information sheet has been fully explained. I understand the benefits and risks of this study. My participation in this project is voluntary.

I acknowledge that I have the right to question any part of the procedure and can withdraw at any time without penalty of any kind.

Name of participant

Signature of participant

Name of researcher

Signature of researcher

Date
