# Supplementary material for: Feasibility and preliminary effects of a mindfulness-based physical exercise (MBPE) program for community-dwelling older people with sarcopenia: A protocol for a parallel, two-armed pilot randomised controlled trial
Source: PLoS One. 2024 Apr 18;19(4):e0302235. doi: 10.1371/journal.pone.0302235 (PMC11025830; doi:10.1371/journal.pone.0302235)
Supplement: S3 Appendix — (PDF) [file pone.0302235.s003.pdf]

The utility of HOme practice in Mindfulness-based physical Exercise program (HOME program) for community-dwelling older people with sarcopenia: a pilot randomised controlled trial

Abstract

Background

Sarcopenia is a geriatric syndrome characterised by low muscle mass, muscle strength, and physical function. Physical exercise (PE), especially resistance exercise, has demonstrated potential effects on sarcopenia. However, low motivation for regular PE is always reported as a major barrier in this population. Based on the PRIME (Plans, Responses, Impulses, Motives, and Evaluations) theory of motivation, Plan (i.e., well-planned, and evidence-based PE protocol), Motives (anticipated pleasure, satisfaction, and relief) and self-awareness on the present moment are the key points of human motivation and the potential targets to change a behaviour (i.e., adhering to a PE intervention) in older people with sarcopenia. Limited study on sarcopenia consisted of any interventional element to emphasise motives and self-awareness on the present moment, two key points of human motivation in the PRIME theory of motivation, although older people with sarcopenia are more likely to have negative feelings (i.e., fatigue, depress, anxiety) and experiences (i.e., derailed by daily life and distracted by concerns on PE). Mindfulness-based intervention (MBI) is a psychosocial intervention which aims to increase the participants' awareness of the present moment non-judgmentally. Increased evidence has indicated that MBI can also improve the levels of PE and adherence to PE interventions. Based on the mindful coping model, the positive relationship between mindfulness and PE might be that MBI has the potential to improve motivation for PE by addressing three key points of human motivation in the PRIME theory of motivation (focusing on the present moment, evoking positive feelings, and increasing self-awareness). Thus, MBI has

the potential to be integrated into PE training to increase motivation and adherence to PE in older people with sarcopenia.

To address the above research gaps, we first conducted a SR on the home-based interventions among community-dwelling older people with sarcopenia was conducted, showing that home-based resistance exercise is feasible, suitable and has potential effects on sarcopenia, but few studies consisted of motivation-enhancing component. Then a Delphi study was conducted to develop the HOme mindfulness-based physical Exercise (HOME) intervention on this population and the evaluation of ender-users (namely community-dwelling older people with sarcopenia) will be adopted to revise this intervention to be more targeted and practical. However, the feasibility, acceptability, and effects of this intervention among community-dwelling older people with sarcopenia need to be explored.

Objective

The objective of this study is to assess the feasibility, acceptability, and the preliminary effects of the HOME intervention among community-dwelling older people with sarcopenia.

Methods

A pilot randomised controlled trial (RCT) will be conducted to assess the feasibility, acceptability, and the preliminary effects of the HOME intervention. In the parallel-group, pilot RCT, 60 community-dwelling older people aged 60 years or older diagnosed with sarcopenia will be randomised into either the intervention group receiving the HOME intervention 2 sessions weekly over 12 weeks or the control group receiving health educations. Each session of the HOME intervention will last about 70 minutes, including 20-min MBI, 40-min HBE (10-min warm-up, 20-min RE, and 10-min cool down) and 10-20-min sharing and discussion. The feasibility of this programme will be determined by time spent recruiting participants, eligibility rate and recruitment rate. The acceptability of the HOME program will be assessed by: 1) prospective acceptability: recruitment rate and reasons for not involving in this study; 2) concurrent acceptability: attendance rate,

complete rate, attrition rate and reasons for discontinuing; and 3) retrospective acceptability: the participants' perspectives on the intervention after taking part. Based on our conceptual framework, primary outcomes (muscle mass, muscle strength and physical function) and secondary outcomes (motivation, depressive symptoms, psychological well-being, mindfulness level and quality of life) will be assessed at baseline (T0), and immediately post-intervention (T1). The quantitative data will be analysed by generalised estimating equations. The qualitative data will be Brun and Clark's thematic approach.

Impact and significance

PE is essential to treat sarcopenia, while the motivation and adherence to PE are major concerns of older people with sarcopenia. Based on the PRIME theory of motivation and the mindful coping model, this study will adopt the mindfulness-based PE (the HOME program), combining the MBI and the conventional PE, which is novel in the research field related to sarcopenia. The HOME program addresses the limitations of previous studies on sarcopenia by integrating MBI into PE to recognise the key points of human motivation in the PRIME theory of motivation, such as self-awareness, positive feelings and focusing on the present moment. This study has the potential to improve the symptoms of sarcopenia, the motivation and adherence to PE as well as the psychological health of this population, which finally improves the holistic welling of this population. For researchers, this study provides a relatively new sub-area in this field by generating insights on the importance of the above factors and the potential effectiveness of the mindfulness-based PE. For health professionals, the study provides a potentially effective way to improve the motivation and adherence to PE to treat sarcopenia.

Introduction

1.1 Prevalence of Sarcopenia

Sarcopenia is defined as a progressive and generalised skeletal muscle disorder that involves the accelerated loss of muscle mass, muscle strength and physical function [1], indicating that sarcopenia is an age-related syndrome and the prevalence in adults aged over 85 years is as high as 64.3% [2]. With the rapid population ageing, sarcopenia is becoming an important health issue for older people globally. Due to the prevalence and significance of sarcopenia, WHO has classified it as an independent disease with the code ICD-10-CM (M62.84) [3]. It is estimated that currently there are more than 120 million adults aged 60 years or over around the world who are sarcopenic, and the number is expected to double to 240 million by 2050 [2, 4]. In China, there are more than 59 million older people aged with sarcopenia [5, 6], which is almost half of older people with sarcopenia in the world.

1.2 Negative impact of sarcopenia

Sarcopenia has brought so much harm and burden on older people, the family, and society. For older people, sarcopenia significantly diminishes their muscle strength and physical function, which further influences their mobility as well as their quality of life. Besides, sarcopenia significantly increases the risks of various adverse health events, such as falling by 3.23 times, functional decline by 3.03 times, fracture by up to 3.75 times [7] and cognitive impairment by 2.85 times [8]. It is also negatively associated with mental health [9] and cardiovascular health [10, 11]. All these negative impacts of sarcopenia in older people will eventually increase the chance of hospitalisation by 2.07 times [12] and even all-cause mortality by 2.20 times [13]. For families, the burden of caregivers or family members will significantly increase as older people with severe levels of older people with sarcopenia rely on someone to care for their daily living [14]. The caregivers or family members need to take care of their daily life, be emotionally supportive and manage the symptoms of sarcopenia, which might greatly

aggravate their strain, anxiety, and stress [15]. For society, a review including 14 studies has indicated that this condition is positively associated with increased healthcare costs and medical resources [16], which considerably exacerbates the shortage of healthcare professionals and resources. Thus, effective interventions are needed to delay or even reverse the progress of sarcopenia to reduce the incidence of various health adverse outcomes, decrease the health cost as well as improve the quality of life.

1.3 Physical exercise (PE) and Sarcopenia

1.3.1 The significance of PE for sarcopenia

Sarcopenia is a complex condition, and the major causes are hormonal changes, chronic inflammation, nutritional deficiencies and particularly physical inactivity and sedentary lifestyle [17, 18]. Declined physical exercise (PE) is one of the key factors in the development of sarcopenia [1, 19]. It is well-known that even 5 days of physical inactivity can lead to muscle atrophy [20]. Conversely, regular PE can reduce the risk of sarcopenia [18] and is strongly recommended as the first-line treatment for sarcopenia [21]. Many studies have shown that PE is effective in improving the symptoms (i.e., physical function, muscle strength) of older people with sarcopenia [22-26].

1.3.2 The limitations of PE interventions on older people with sarcopenia

Although previous studies support the benefits of PE on sarcopenia, limited studies fully consider the characteristics and preferences of this population, such as staying at home due to low mobility and physical function [27]. A previous study has also indicated that older people prefer doing exercise in a familiar environment, such as home, than in a formal group setting [28]. However, the effectiveness and feasibility of home-based interventions on sarcopenia are still inconsistent [29], details in Chapter 2.

Furthermore, although evidences have shown interventions (i.e., exercise, nutrition supplement) can improve the symptoms of sarcopenia (i.e., physical function, muscle strength) [22-26], they included the older people with sarcopenia

living in various settings (i.e., nursing home, hospital, community)It is important to note that older people in various settings have considerable differences in terms of health backgrounds, levels of ability, and factors on interventions [30, 31]. For example, older people in nursing homes are more likely to be malnourished and depressed, which could influence their adherence to PE intervention. While the biggest issue for older people, mainly those with frailty and sarcopenia, living in the community, is less physical activity [32] and major barrier to PE is low motivation (i.e., lack of supervision, well-planned program) [33]. Thus, it is necessary to identify an effective intervention for improving the sarcopenia of community-dwelling older people.

More importantly, low adherence to regular PE is a major concern in older people with sarcopenia, especially in home-based intervention with limited monitoring [34]. A systematic review (SR) has demonstrated that the adherence of home-based exercise (HBE) in healthy older people is 58% for resistance exercise (RE) and 63% for walking [35]. The adherence of older people with sarcopenia might be lower due to the low mobility and physical function [36]. Although adherence is influenced by many factors (e.g., physical function, supervision, perceived benefits), according to the PRIME (Plans, Responses, Impulses, Motives and Evaluations) theory of motivation, motivation is the most fundamental and direct factor for a behaviour (engaging in and maintaining a PE intervention) as other factors impact on the behaviour via motivation [37, 38]. In addition to the PRIME theory of motivation, studies have repeatedly reported that lack of motivation is a main concern on participating in and maintaining PE in older people with sarcopenia [33, 39-41]. For example, a study has illustrated that older people with sarcopenia just feel not motivated to exercise [42]. Thus, how to activate the motivation of older people with sarcopenia is crucial and potentially the most effective way to improve the adherence to PE interventions.

The COVID-19 pandemic also has greatly decreased the levels of PE in older people with sarcopenia due to the lockdown of parks, sports facilities, and

gymnasiums [43, 44]. In addition, social isolation or home quarantine leads to great reductions in PE and increases in sedentary behaviour [45, 46], which significantly accelerates the deterioration of sarcopenia [1]. HBE program would be a solution for these barriers and preferred by older people with sarcopenia.

1.4 Motivation for PE

In order to better understand behaviour change, the PRIME theory of motivation proposes that the human motivation system operates at five levels, from the top to bottom, plans (intentions to change), evaluations (beliefs about what is good or bad), motives (anticipated pleasure or satisfaction or relief), impulses (urges) and response [38], **Figure 1**. From the five levels of human motivation systems, the behaviour change is a moment-to-moment response (motivation) by evaluating whether the plan to change (Plan) is aligned with their beliefs (Evaluation) and more importantly whether can generate strong positive feelings (Motives). As indicating by this theory, intensions or beliefs are just stimuli and can activate motivation only when they generate positive feelings or thoughts [47]. In terms of PRIME Theory, to change a behaviour (i.e., adhere to PE intervention), it involves the first three levels of human motivation system: 1) Plan: forming a plan or rule to change (i.e., the evidenced-based PE protocol), 2) Evaluation: continuously enhancing the participants' beliefs in the benefits of the plan (i.e., the benefits of PE), and 3) more crucially, Motive: activating the positive feelings about the changing process (i.e., the pleasure, satisfaction, or relief during PE). Besides, this theory emphasizes the importance of self-awareness at the present moment, indicating that self-awareness, focusing on ourselves, is a powerful source of pleasure and the initial step of self-control [48]. In summary, based on the PRIME theory of motivation, the potential targets to activate the motivation to PE in older people with sarcopenia are a well-planned and evidence-based PE protocol (Plan), strengthening the beliefs in the benefits of PE (Evaluation), eliciting positive feelings towards PE (Motives), and self-awareness on the present moment.

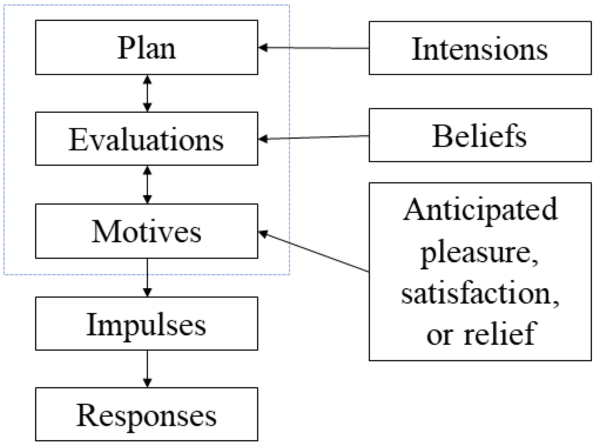

Figure 1 The PRIME theory of motivation [38]

The benefits of PE on health (beliefs) are commonly recognised by older people [49], even older people with sarcopenia [50]. Thus, it is potentially the other three factors (Plan, Motives, and self-awareness on the present moment) are essential to improve the motivation for PE in older people with sarcopenia. The PE protocol used in this study is evidenced-based to provide a detailed plan for older people with sarcopenia as it is developed by conducting a SR **(Supplementary 1)**, referring to guidelines, reviewing by experts via Delphi technique **(Supplementary 2)**. As for the other two potential targets, positive feelings towards PE (Motives) and self-awareness on the present moment, we found that limited studies on sarcopenia explore them although older people with sarcopenia are more likely to have negative thoughts and feelings, such as feeling fatigue or discomfort while doing exercise [27], feeling sad or hopeless when they think they are unable to do the intended PE or anxiety when they are eager to see the effectiveness of PE [39] due to decreased muscle mass, low physical function and low exercise ability. Besides, older people with sarcopenia are easily derailed by issues in daily life [51] and distracted by their preconceived misunderstandings towards PE, such as being too old and too dangerous to perform any PE [33], which significantly decrease their attention on the PE tasks and thwart their motivation for PE, based on the PRIME theory of motivation. All these negative feelings and experiences are also likely to undermine their psychological well-being and even

cause mental issues (i.e., anxiety, depression) [52]. It is well-known that there is a vicious cycle between mental issues (i.e., depression) and PE , where depressive symptoms have a great impact on a person’s thoughts, feelings and motivation [53, 54], such as motivation for PE [55]. Therefore, a vicious cycle between sarcopenia and low motivation for PE (**Figure 2**) to further explain why this population has low motivation and how it further deteriorates the development of sarcopenia.

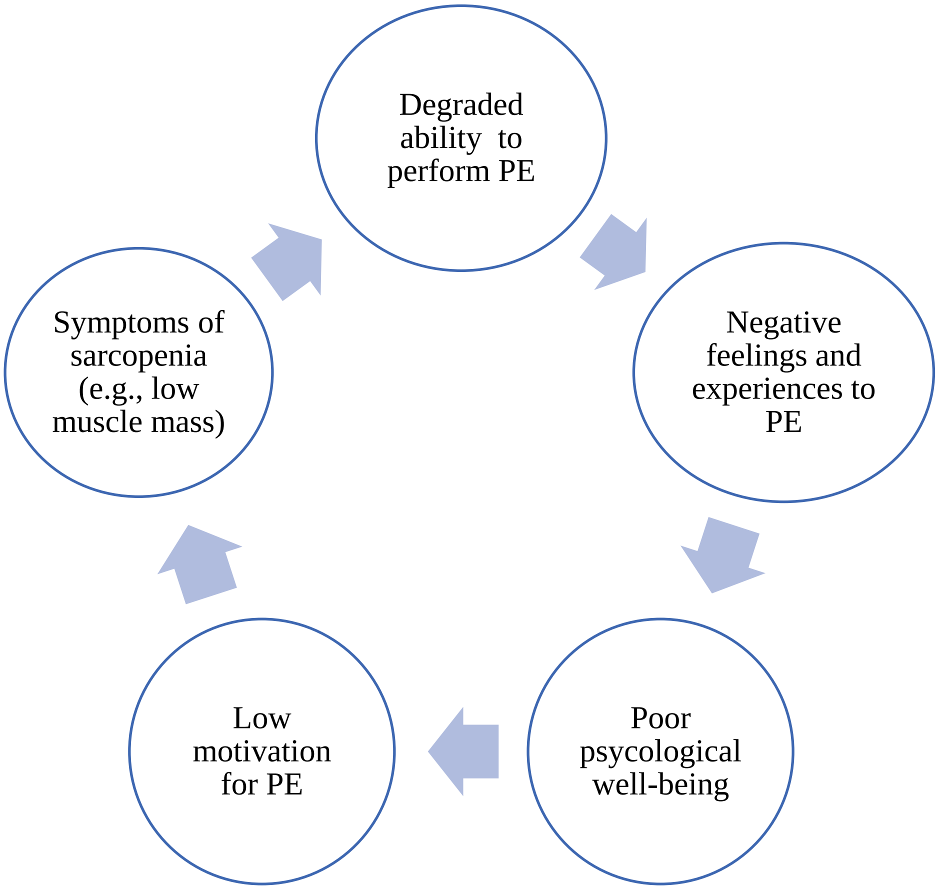

Figure 2 Vicious cycle between sarcopenia and low motivation

Therefore, based on the PRIME theory of motivation and the vicious cycle **(Figure 2)**, in addition to a well-planned and evidence-based PE protocol (Plan), a way to eliciting positive feelings (Motives) and self-awareness on the present moment are highly required to improve the motivation for PE and the holistic health of older people with sarcopenia.

1.5 Mindfulness and PE

Mindfulness comes from Buddhism but is tested in scientific literature, and defined as a process of openly attending, with awareness, to one’s present moment

experience [56]. The aim of mindfulness-based interventions (MBI) is to increase self-awareness at the present moment and enhance a non-judgmental mind. MBIs have shown promising effects on various health issues. It is regarded as the "third wave" of psychological therapy because it effectively reduces depression, anxiety, and stress [51, 57]. Besides, MBI is also an efficacy way to reduce pain [58], improve quality of sleep [59], cognition [60] and quality of life [61].

In addition, more and more studies found that mindfulness is associated with higher levels of PE [62-64]. For example, a SR, including 20 cross-sectional studies, showed a positive relationship between mindfulness and PE [65]. This SR also including 20 interventional studies on MBIs, demonstrates a beneficial effect of MBIs on PE [65]. Similarly, another SR including 13 studies, indicated a positive association between mindfulness and PE [66]. Besides, a 4-week RCT has found that mobile phone-based MBI (audio-recorded mindfulness text message) is also acceptable and effective in improving PE and activating the motivation for PE in older people [67]. Moreover, MBIs can potentially improve the effectiveness of interventions and adherence even after the intervention is terminated [68]. The positive relationship between mindfulness and PE might be explained by the mindful coping model via improving the motivation for PE by addressing three key points in human motivation in the PRIME theory of motivation (details in Chapter 3, 3.4). As mentioned before, based on the PRIME theory, in addition to well-planned and evidence-based PE protocol (Plan), anticipated pleasure, satisfaction or relief (Motives) and self-awareness on the moment are essential to improve motivation for PE in older people with sarcopenia. MBI can improve motivation by addressing these three key points of human motivation based on the mindful coping model. First, being at the present moment by using MBI may enable participants to focus on relevant PE tasks, not be derailed by other factors, such as issues in daily life [69], which emphasises the importance of focusing on the moment on human motivation in the PRIME theory of motivation. Second, the non-judgmental way, a crucial element of MBI based on the mindful coping theory,

would enhance the acceptance of negative or uncomfortable thoughts and sensations (e.g., pain, fatigue, and exertion) [66, 70]. This is the core stone of self-awareness and the first step for relief from stress and anticipated pleasure (Motives), which are two essential points of human motivation in the PRIME theory of motivation. Moreover, based on the mindful coping theory, the positive reappraisal and appropriate emotion response evoked by MBI would improve participants' satisfaction and enjoyment during exercise [64, 71],which is the most vital part of human motivation based on the PRIME theory of motivation.

In summary, MBI can be used to improve the motivation for PE based on the mindful coping model by improving three key points of the human motivation in the PRIME theory of motivation: focusing on the moment, accepting of negative feelings (Self-awareness and Motives), and evoking positive feelings (Motives). Besides, MBI can improve psychological health to further enhance the motivation for PE based on the vicious cycle between sarcopenia and low motivation **(Figure 2)**.

1.6 Delivery modes of physical exercise program

As we mentioned before, HBE intervention is more convenient, and feasible in different situations (e.g., during a pandemic). There are mainly three delivery modes of physical exercise programs which are: face-to-face teaching, self-directed learning, and e-health intervention [72-74].

1.6.1 Face-to-face teaching

There are several modes of face-to-face health intervention, such as group-based interventions at the healthcare centre, gym or research setting, and door-to-door interventions [72]. While only the latter one is workable in the home environment. Door-to-door health intervention has several advantages. First, it greatly increases the possibility that participants learn and carry out the content of health intervention the same as the intended protocol, especially for exercise intervention [75]. This is because researchers could teach participants the health intervention program and to correct them if they don't understand or do the

program wrongly. Therefore, this mode of delivery is capable of improving the intervention fidelity by improving intervention receipt [76]. Furthermore, it is more feasible and convenient to have tailored, individual intervention programs in door-to-door mode [77], which enables researchers to have more detailed health assessments of each participant, such as the physical fitness, health literacy, and lifestyle before and during the health intervention. After detailed health assessments, researchers can make and adjust the health intervention more personally to have a better beneficial effect. However, this mode does have some challenges. The biggest one is the high demand of health professionals to keep this type of health intervention going as one or two health professionals cannot carry out and finish such heavy work. While the lack of health professionals is a universal problem [78]. There is another problem emerging if many health interventionists are involved in one project, the consistency [76]. It might be difficult to deliver the exact same program protocol as the intended one because it is conducted by so many different interventionists, which considerably reduces the intervention fidelity [79].

1.6.2 Self-directed learning from health promotion materials

There are several types of self-directed learning suitable for the home environment, including reading in newspapers, booklets, brochures, and leaflets [74]. This mode of delivery is portable, which means the participants can learn and use it anywhere they like [80]. For example, they can learn about the health program even on the road to buy groceries. However, participants only passively received health intervention programs through this one-way delivery mode. They might be unable to understand the program content or carry out the program as the intended one due to a lack of mutual communication between participants and researchers [34]. This is especially true for older people whose learning capacity is greatly degraded. All the issues might result in the inaccuracy of the health program (i.e., exercise movements) in older people and thus undermine the efficacy of the self-directed learning health programs in the home environment

[34]. More importantly, the interest and initiative in the learning of older people has also decreased, which might result in low adherence to self-directed learning interventions [81, 82].

1.6.3 Electronic-health intervention

The rapid growth of technology has made a great impact on the way we communicate, interact, learn, and work. In health care, electronic-health, known as e-health, defined as the health interventions conducted through the internet and related technologies (i.e., mobile phone, tablet, website), has penetrated almost all aspects of the health care system and health interventions [83]. In Mainland China, websites, social media, mobile applications, wearable devices, telephones are commonly used for e-health interventions in health care [72]. E-health intervention has several advantages compared to other modes of delivery. First, it provides us a chance to realise cross-time, cross-region and cross-distance communication, effectively break down information barriers, and promote information transmission and sharing [80]. This is particularly important in the COVID-19 pandemic for home-quarantine or social isolation is still required in some areas of mainland China. Besides, the common characteristics of e-health technology also make it popular in health interventions, such as in-time communication and feedback, interactive, enjoyable and engaging intervention program as well as reinforced environment via some e-health technology (i.e., virtual reality, exergame) [83, 84]. These characteristics of e-health technology would activate motivation of participants to participate and maintain the health intervention. Moreover, some e-health interventions can be individually tailored and progressed by participants themselves and easier to self-monitor via tracking their own progress [85].

Despite all the merits of e-health intervention, it does have some disadvantages. The biggest one is the difficulty of using technology in older people [84]. And this might have two reasons: one is that some e-health interventions are indeed quite complex because designers did not fully consider the characteristics

of older people, such as degraded ability of understanding and learning, poor eyesight [73]. The other reason might be the fear and resistance of older people to e-health technology, especially to these apps, websites, or other materials that they are not familiar with. Older people, particularly those with low digital health literacy, might have such negative feelings even before using new e-health technology. Therefore, choosing a technology or platform that is already used and familiar by older people is the optimal way to avoid these negative feelings.

Among all used e-health technology in health interventions, the social media platform is widely accepted as an important health intervention tool due to its capability to disseminate information efficiently and facilitate communication in China [86]. In particular, WeChat, a social media platform launched in 2011, has obtained rapid and enormous popularity among Chinese people [86]. It is estimated that WeChat has more than one billion active users globally, including 63 million aged 55 or above [87]. Similar to other social media platforms (i.e., Facebook, Twitter), WeChat is a free instant messaging application for smartphones, tablets, and laptops, which enables its users to share text, photo, voice, video and other media-messages (i.e., link, mini-program) as well as to make voice/video-calls [88]. Besides, up to 500 people can have in-time communication simultaneously by making voice-calls via WeChat Group, a virtual chat group in WeChat [89]. WeChat has greatly influenced dissemination of information across the areas of healthcare and is a powerful tool for health education and intervention. It shows promising effects on various health managements, such as chronic disease management [90], psychological well-being [91, 92] and smoke cessation [93]. Studies also demonstrate that WeChat-based intervention is well-accepted in middle-aged and older people [94, 95] and improves physical activity [95].

Therefore, the WeChat platform might be the most feasible and acceptable solution to conduct home-based intervention in Chinese older people with sarcopenia as it is well-accepted by this population. However, the actual way of

delivering the intervention will depend on the real situation of our participants, such as their familiarity of using WeChat.

1.7 Summary

Sarcopenia is a common geriatric syndrome worldwide due to rapid ageing, causing various health adverse outcomes, which burden older people, the family, and society. PE is the priority way to treat this disease and shows beneficial effects on some symptoms of sarcopenia (i.e., muscle strength, physical function). Home-based interventions are more convenient, preferred, and practical for this population due to their low mobility and unwillingness to go out, especially during the COVID-19 pandemic. While low motivation leading to high dropout is a major barrier to home-based intervention. Based on the PRIME theory of motivation, Plan (i.e., well-planned and evidence-based PE), Motives (anticipated pleasure, satisfaction, and relief) and self-awareness on the present moment are the key elements of human motivation and the potential targets to change a behaviour (i.e., adhering to a PE intervention) in older people with sarcopenia. Although older people with sarcopenia are more likely to have negative feelings (i.e., fatigue, depress, anxiety) and experiences (i.e., derailed by daily life and distracted by concerns on PE), there are limited study on sarcopenia containing any interventional element to emphasise these issues. These negative feelings and experiences directly undermine their motivation for PE based on the PRIME theory of motivation and by influencing their psychological health based on the vicious cycle between sarcopenia and motivation **(Figure 2)**.

Mindfulness is a process of openly attending, with awareness, to one's present moment experience. MBIs have shown promising effects on various health issues, including depression, pain conditions, smoking, addictive disorders, stress, sleep and eating. Moreover, increased evidence has implied that MBI is associated with higher levels of PE and better adherence to PE interventions. Based on the mindful coping model, MBI improves the levels of and adherence to PE is potentially through activating motivation for PE via enhancing three essential points of human

motivation in the PRIME theory of motivation: focusing on the present moment, accepting of negative feelings (Self-awareness and Motives), and evoking positive feelings (Motives). Thus, mindfulness is not only able to improve psychological well-being to further enhance motivation for PE, but more importantly, can be used to directly improve the motivation and adherence to PE.

There are several modes of delivery of health interventions in the home environment in mainland China, including door-to-door, traditional self-learning materials and E-health based intervention. Among those, social media, in particular WeChat, has been widely used in healthcare interventions and accepted by older people. Therefore, WeChat platform will be adopted in our study.

From the literature, we have identified:

What is already known?

1) PE is a priority intervention for older people with sarcopenia to improve the symptoms of sarcopenia (i.e., decreased muscle mass, physical function, and muscle strength).
2) Home-based intervention is more convenient, preferred, and practical for this population, especially during the COVID-19 pandemic. While low motivation leading to poor adherence is a major barrier especially to home-based interventions.
3) Based on the PRIME theory of motivation, Plan (i.e., well-planned and evidence-based PE), Motives (anticipated pleasure, satisfaction, and relief) and self-awareness on the present moment are the potential targets to behaviour change (i.e., adhering to a PE intervention) in older people with sarcopenia.
4) MBI can be used as an adjunction to exercise to improve the motivation and adherence to PE through addressing three essential points of human motivation in the PRIME theory of motivation: focusing on the present moment, accepting of negative feelings (Self-awareness and Motives) and evoking positive feelings (Motives), based on the mindful coping

theory.

5) A home-based intervention consisting of both physical and motivation-enhancing components is strongly required to improve the symptoms of sarcopenia and motivation for PE.

What is still unknown?

1) The effectiveness and modes of delivery of home-based PE interventions in this population are still unclear.
2) Whether the current home-based PE intervention in older people with sarcopenia consists of motivation-enhancing components is not known.
3) There is limited study on mindfulness-based PE in older people, particularly those with sarcopenia. The feasibility and effectiveness of integrating MBI in home-based PE are not well-known.
4) Whether the motivation and adherence to PE can be improved by home mindfulness-based PE program is needed to explore.

To answer these questions, a SR on the home-based interventions on community-dwelling older people with sarcopenia was conducted **(Supplementary 1)**, showing that home-based resistance exercise is feasible, suitable and has potential effects on sarcopenia, but few studies consisted of motivation-enhancing component. Then a Delphi study was conducted to develop the HOme mindfulness-based physical Exercise (HOME) intervention on this population **(Supplementary 2)**. However, the feasibility, acceptability, and effects of the HOME intervention among community-dwelling older people with sarcopenia need to be explored.

1. Objectives and hypotheses

1.1 Aim and objectives

The aim of this trial is to evaluate the feasibility and acceptability as well as the preliminary effect on physical and psychological health of the HOME program in older people with sarcopenia in the community. Thus, the objectives of this trial are:

1) To assess the feasibility and acceptability of the HOME program among community-dwelling older people with sarcopenia, in terms of
   a) Feasibility of participant recruitment: time spent recruiting participants, eligibility rate and recruitment rate of the participants
   b) Acceptability of the HOME program in community-dwelling older people with sarcopenia: prospective, concurrent, and retrospective acceptability
2) To explore the preliminary effects of the HOME program on the following outcomes:

   Primary outcomes

   The symptoms of sarcopenia (skeletal muscle mass index (SMI), handgrip strength and physical function)

   Secondary outcomes

   a) Motivation
   b) The level of mindfulness
   c) The psychological well-being
   d) The depressive symptoms
   e) Quality of life

1.2 The hypotheses

The hypotheses of this trial are:

1) The HOME program is feasible and acceptable among the community-dwelling older people with sarcopenia.
2) The HOME program will significantly improve the symptoms of sarcopenia, motivation and adherence to exercise as well as the psychological well-being

in the older people with sarcopenia, compared with the control group.

2. Methods

This study will adopt a two-arm pilot RCT design with the 1: 1 ratio nested with individual interviews. The Consolidated Standards of Reporting Trials (CONSORT) checklist for a pilot trial will be followed. The pilot trial is chosen for two reasons: 1) there is limited mindfulness-based PE in older people with sarcopenia; 2) feasibility and pilot trial is a crucial stage for assessing the feasibility and acceptability of the intervention in order to make decisions about the next stage according to the MRC guidance [96]. RCT is chosen because it is regarded as the most rigorous design of interventional studies based on the hierarchy of evidence.

2.1 Study settings

The participants will be recruited from the three community health care centres (CHCC) in Suzhou, Jiangsu Province, Mainland China. These CHCCs provide annual free body examination for older people who are 60 years or older living in the community. An office with ample space in each CHCC will be utilized for screening of eligibility (i.e., body composition test, physical function test) and the outcome measurement at baseline and immediately after the intervention.

2.2 Sample selection criteria

This study focused on community-dwelling older people diagnosed with sarcopenia. The inclusion and exclusion criteria are described in **Table 1**.

Table 1 the eligibility of participants in this study

Inclusion criteria	Exclusion criteria
Are community-dwelling people aged 60 years or older	Have been hospitalized for more than 5 days in the preceding 3 months
Are diagnosed with sarcopenia by the criteria of the Asian Working Group for Sarcopenia (AWGS) [19]:<br>1) decreased muscle strength: handgrip strength of males is less than 28 kg; handgrip strength of females is less than 18 kg;<br>2) decreased physical performance: gait speed of 6 meters is less than 1.0m/s;<br>3) decreased muscle mass: SMI of males is less than 7.0 kg/m^2; SMI of females is less than 5.7 kg/m^2<br>Diagnosis of sarcopenia: meet criteria 3 and 1 or/and 2	Are unable to have body composition test, such as having heart pacemaker, vascular stent, steel plates and nails in the body
Are able to communicate and written and understand the instruction	Have contraindications to exercise, such as severe musculoskeletal disorders, severe cardiovascular diseases or spinal nerve injury
Have a WeChat account	Have regular exercise: 150-minute moderate-intensity activity or 75-minute vigorous-intensity activity per week, with each session lasting at least 10 min in the past 3 months based on self-reported time and a self-perceived intensity via Borg Scale[97, 98]

	Practice mindfulness/yoga for >45 min a week in the 6 months prior to recruitment [99]
	Have considered impaired cognition as indicated by the Abbreviated Mental Test score < 7 (sensitivity of 92.3% and specificity of 87.1%) [100]

2.3 Sample size estimation

The pilot RCT with a sample size of 25 per group will produce at least a small standardized effect size in the main study [101]. Besides, an attrition rate of 20% will be assumed [102], thus the sample size of this pilot RCT is $25 \times (1 + 20\%) = 30$ in each group. A total of 60 participants are needed. This is also in the range of sample size (from 10 to 40 per group) suggested by Hertzog [103].

2.4 Recruitment procedure

Convenience sampling method will be used to recruit participants in this study. Every older people coming to the CHCCs will be invited to participate in the screening of this study by trained research assistants after informed consent. The procedure of the screening will first ask older people for basic information (name, age, whether they have heart pacemaker) to decide whether they can have the tests of sarcopenia diagnosis as well as to assess their weight and height to prepare for the body composition. Then, the sarcopenic status of older people will be assessed based on the latest sarcopenia criteria and procedure of AWGS (details in **Figure 3**). The older people with sarcopenia will be invited to give more information about their eligibility and willingness to this study via telephone or face-to-face within three days after the diagnosis of sarcopenia. The participants will also be recruited via telephone, WeChat, and mouth of words.

After the participants recruitment, the eligible participants will be approached by trained research assistant to explain this study in detail (i.e., the purpose, intervention, potential benefits, and risks). The information sheet will be given to the participants and written informed consent will be obtained.

2.5 Randomization and Allocation Concealment

After informed consent and baseline measurements, eligible participants will be randomized into intervention group (IG) or control group (CG). The block randomization with block size of 4 will be used to guarantee balance in numbers in each group. Three research assistants (trained post-graduate nursing students) who won’t participate in any session of this study will perform the randomization

independently to keep allocation concealment and to avoid the selection bias. Specifically, a research assistant (A) will prepare a list of the participants, assign a code for each of them and code the intervention and control group as group A or B. The other research assistant (B) will code the possible six orders of the blocks (i.e., 1 is AABB, 2 is ABAB) and randomize the code by using a computer-generated random number and keep the number in an opaque envelope. After these, research assistant C is to conduct the random allocation to participants by opening the envelope only when the number of participants in at least one block (4, 8, 12 participants) are all available to avoid the non-randomization of the last person in the block.

2.6 Blinding

Due to the character of the intervention and control group in this study (the mindfulness-based PE vs usual care), it is impossible to blind participants. The interventionist (the qualified mindfulness instructor and sport coach) is also unrealizable to be blinded. To reduce the allocation bias as much as possible, the following personnel will be blinded: the research assistants who are responsible for the participants recruitment, the research assistants who perform the randomization and the assessor who collect data at baseline, week 8 and immediately after intervention. The health professionals in the CHCCs won't be involved in this study, only providing some basic help, such as materials (tables, papers).

2.7 Intervention

After baseline measurement, the participants will be randomized into IG or CG for 12 weeks. The intervention in each group is described below:

3.7.1 Intervention group

Participants in the IG will receive the HOME program. Generally, the participants will receive about 70-min HOME program twice a week. The intervention will include 1) introduce the theme of this session, 2) mini lectures about the theme; 3) mindfulness practice related to the theme; 4) tips of keeping

mindfulness during PE; 5) warm up: lead with mindfulness words; 6) resistance exercise; 7) cool down: lead with mindfulness words; 8) discussion. The HOME intervention will be conducted by a qualified mindfulness instructor, who is also familiar with physical exercise, with the companion of a qualified sport coach. All the lessons will be recorded to facilitate the participants review if they want as well as to help check the intervention fidelity. The participants will be required to write the intervention blog dairy to check their adherence and compliance. Telephone follow-ups at week 2, 4, 7 will be used to check whether the participants have any questions or difficulties while participating in this program. Home-visit will be utilized if the participants meet any questions that cannot be solved through remote supervision (e.g., telephone call, WeChat video). Besides, homework (walking 20-30 min and other mindfulness activities) after each class will be given to the participants to maximize the benefits of the HOME intervention. The actual way of delivering this intervention will be determined by the familiarity of the participants on using of WeChat.

3.7.2 Control group

The aim of control group is to avoid the potential factors on the effectiveness of the primary outcomes. In this study, as the primary outcomes are objective indicators (muscle mass, muscle strength and physical function), the only potential factor is level and habit of PE of the participants. This is considered in the inclusion and exclusion criteria as those older people with sarcopenia with regular exercise will be excluded in this study. In addition, socialization and interaction might influence the motivation and adherence to the HOME program. Thus, health education consisting of discussion provided by a registered nurse will be conducted as a control of socialization and interaction. The number of sessions, duration, frequency, group size and delivery modality will be similar to the intervention group. The topics of the health education are the care of common diseases in the older adults, including hypertension, diabetes, osteoporosis, COPD, dementia, depression. Each session is about 70 min (same as intervention group),

including 10-20 min lecture and 50-60 discussion and sharing.

Considering ethics and avoiding attrition, the participants in the CG will be promised to receive the video of the online lessons taught in the IG. For contamination, because participants will be recruited from different communities, the chance of knowing each other and communication will be extremely low. If two or more participants indeed know each other, then only one of them will be included in this study by draw lots before baseline assessment.

3.8 Outcomes and measurements

In addition to socio-demographic information, the primary outcomes of this pilot RCT are the feasibility and acceptability. Secondary outcomes are the preliminary effects on the symptoms of sarcopenia, depressive symptoms, psychological well-being, physical activity level, mindfulness level and quality of life. The socio-demographic information will be collected only at baseline (one week before the intervention, T0). All the outcomes will be evaluated at T0, and immediately after the intervention (T2) by a trained research assistant, who are independent assessors and blinded to the randomization. The data and measurements are described below.

3.8.1 Socio-demographic information

The socio-demographic information includes three parts: the demographic information (i.e., name, age, marital status), general information (i.e., disease history, injury history, medication) and information on exercise.

3.8.2 Feasibility

The feasibility will be evaluated by the following outcomes: the time spent recruiting participants, eligibility rate (the number of eligible participants/the number of screened participants) and recruitment rate (the number of participants recruited/ the number of eligible participants).

3.8.3 Acceptability

Sekhon et al [104] suggest that acceptability is multi-faceted and have proposed a theoretical framework of how to measure acceptability, including

prospective acceptability (prior to the intervention), concurrent acceptability (during the intervention) and retrospective acceptability (after the intervention). These three constructs will guide the assessment of acceptability of this pilot RCT.

(1) Prospective acceptability

Prospective acceptability is about the acceptability of the participants prior to participating the study. It is determined by affective attitude (what are the participates' feelings about the intervention before participating) and burden (reasons for not engaging in the intervention) [104]. Thus, in this pilot RCT, recruitment rate will be used to measure the affective attitude while reasons for not involving in will be given by the older people to assess burden.

(2) Concurrent acceptability

Concurrent acceptability is about the intervention adherence (the involvement in the intervention of participants) and coherence (the extension of participants understands the intervention) [104]. In this study, these will be assessed by attendance rate (attended sessions of the participants/ all online sessions), complete rate (online sessions that actually involved and finished at least 80% / all attended sessions), attrition rate (the number of participants dropped out total number of participants) as well as the reasons for discontinuing. If the participants engage in less than 80% of online sessions will be considered as attrition.

(3) Retrospective acceptability

Retrospective acceptability is mainly about the participants' perspectives on the intervention after taking part, such as the self-perceived benefits, barriers, and satisfactory of this intervention [104]. Thus, this will be explored by individual interviews in this pilot RCT. The individual interviews will be conducted by the PhD student who has received rigor trained in qualitative study methods and engaged in individual interviews before and a professor who has rich experience in conducting individual interviews and is familiar with sarcopenia and mindfulness. The reflective journals written by the

interviewers will be used to avoid bias and keep the transparency of the research process [105]. A research assistant (a master student of nursing with experience of engaging in individual interviews) will help to take notes. All participants in the IG will be invited to join the individual interviews. After the consent, the individual interviews will be carried out face-to-face and audio recorded. The individual interviews guide is described in **Table 3**.

Table 3 The guide open question of individual interviews for participants in intervention group

Number	Questions
Q1	**Generally, what do you think of this intervention?**<br>***Prompts:***<br>-Can you share your feelings of this intervention?<br>-What impact do you think this intervention will bring to your life?
Q2	**What do you think of the content of this intervention?**<br>***Prompts:***<br>-Which is your favorite part of this intervention? Why?<br>-Which do you dislike? Why?
Q3	**What do you think of the frequency, duration and delivery mode of this intervention?**<br>***Prompts:***<br>-How long of the intervention do you think is suitable for you? Why?<br>-How often of the intervention do you think is acceptable for you? Why?<br>-What do you think of the delivery mode of this intervention?
Q4	**How about your motivation to exercise?**<br>***Prompts:***<br>Is it increased? How and why?<br>Are you feel confident to do exercise?
Q5	**What recommendations do you have for this intervention?**<br>***Prompts:***<br>-What do you think should be added? Why?<br>-What do you think should be revised or deleted? Why?

3.8.4 The preliminary effects

The effects of the HOME program on symptoms of sarcopenia (muscle mass, muscle strength and physical function), depressive symptoms, psychological well-being, mindfulness level and quality of life will be preliminarily explored in this study. All the same measurements will be performed by one trained research

assistant to avoid the error between different assessors.

(1) The symptoms of sarcopenia

The symptoms of sarcopenia include low muscle mass, muscle strength and physical function. The detailed outcomes and measurement of each is described as below:

Muscle mass:

Muscle mass will be assessed by SMI, appendicular skeletal muscle mass (ASM), fat-free body weight (FFW), total skeletal muscle mass (TSM) by using Bioelectrical impedance analysis (Inbody 270, Korea) . The procedure of testing is below: before the test, the participants will be required to remove every item that might influence the test, such as metal keys, bank cards, mobile phones, from their body, and then take off their shoes and socks. Then, they will be instructed to stand straight on the detector with two hands holding the handles of the detector for about one minute. During the testing, they will be not allowed to talk, move, or laugh.

Muscle strength:

The indicator of muscle strength is handgrip strength in this study. Handgrip strength will be measured by Jamar dynamometer (Jamar, 563213, USA) and the method refers to the recommendation of American Society of Hand Therapists [106]. Generally, the participants will be required to sit on the chair and rest for at least 5 min before test. The body trunk of the participants needs to keep upright with the shoulder joint in the neutral position and maintaining 90 ° with the elbow joint. During the test, the participants will be instructed to hold the dynamometer in their hands as hard as possible for 3 seconds. The left and right hands will be tested for 3 times separately, with an interval of 30 seconds each time. If the coefficient of variation (CV) is greater than 10, the participants will be asked to rest at least 20 min before testing again. In this study, the maximum value of dominant hand grip strength will be utilized as the result.

Physical function

Physical function will be evaluated by 6-meter gait speed and Time-Up and Go Test (TGUT). The usual walking speed of 6-meter walking test will be assessed. The participants will be instructed to walk a straight-line with a total distance of 10 meters at their usual pace twice with the time of walking the middle 6 meters recorded to avoid bias. For TUGT, participants will be required to sit on a chair with the height of 43 cm before the test. Then they will be instructed to stand up and walking a straight-line with 2.45 meters as fast as they can twice. The mean of the two tests will be adopted in the study.

(2) Motivation for exercise

The motivation to exercise will be evaluated using the Chinese version of the Behavioural Regulation in Exercise Questionnaire-2 (C-BREQ-2). The C-BREQ-2 is a self-report measure with 18-items, containing five domains: amotivation, external regulation, introjected regulation, identified regulation, and intrinsic regulations. Each item is rated on a 5-point Likert scale from 0 "not true for me" to 4 "very true for me". The C-BREQ-2 has been tested in Chinese people and shown good consistency (Cronbach's $\propto$: 0.72-0.83) [107].

(3) The depressive symptoms

Depressive symptoms will be measured with Chinese version of short form Geriatric Depression Scale (GDS-15). GDS-15 is self-rating depression scale specifically for older people developed based on GDS-30 [108, 109]. GDS-15 has been widely used in older people due to its convenience and accuracy [110-112]. It has been tested in Chinese older people and shown acceptable reliability and validity (Cronbach's α:0.793, re-test reliability: 0.728) [113]. The score of GDS ranges 0-15 with each "no" answers of item 1,5,7 and 11 scoring 1 while the rest items scoring 1 with "yes" answers. The classification of depressive symptoms based on the score is: score 0-4: normal; score 5-8: mild depression; score 9-11: moderate depression; score 12-15: severe depression [114].

(4) The psychological well-being

The psychological well-being will be measured with Chinese version of Raff's Psychological Well-being Scale (RPWS-C) which was adapted by Tan [115]. The original RPWS was developed by Ryff el at. including three versions: 84-items, 54-items, and 18-items. It was modified into Chinese and tested in Hong Kong population by Cheng and Chan [116]. While the Cronbach's ∝ was low and there are cultural differences between Hong Kong and mainland China. Thus, the adapted version by Tan [115] will be used in this study. Same as the original version, there are six dimensions in this version, including autonomy, positive relationship with others, personal growth, environmental mastery, purpose in life and self-acceptance. The Cronbach's ∝ of this version is 0.89 and re-test reliability is 0.90.

(5) Physical activity level

Physical activity level will be evaluated by the Chinese Version of the Physical Activity Scale for the Elderly (PASE-C) [117]. The PASE-C, consisting of 10-items, is a self-reported scale to measure the occupational, household, and leisure activities for the last seven days. The PASE-C has been shown good reliability (intraclass correlation coefficient was 0.81) [118]and validity (fair to moderated association with energy expenditure, walking steps and handgrip strength) [119].

(6) The mindfulness level

Mindfulness level of the participants will be assessed by the Five Facet Mindfulness Questionnaire (FFMQ). The FFMQ is a self-rated scale developed by Bear el at. in 2006 [120]. It includes 39 items evaluating five facets: observing, describing, acting with awareness, non-judging, and non-reacting. The Chinese version was firstly introduced by Deng in 2011(FFMQ-39-C) [121] and then was simplified and adapted into Chinese culture by Hou in 2014 (FFMQ-20-C) [122]. However, the two versions of FFMQ has some limitations when using in people in mainland China [123]. The translation of

some items of FFMQ-39-C is difficulty to be understood by Chinese people, such as Item 9 (“*I watch my feelings without getting lost in them*”) [123]. The FFMQ-20-C was validated in people in Hong Kong who might have different cultural background with people in mainland China [123]. Thus, Zhu el at. [123] have proposed an adapted and simplified version (FFMQ-15-C) targeting on people in mainland China with acceptable reliability (Cronbach’s ∝ of all the five facets: ≥ 0.73). The FFMQ-15-C will be adopted in this study with the permission of the author.

(7) The quality of life

Quality of life will be measured with the questionnaire targeting on older people with sarcopenia, Sarcopenia and Quality of life (SarQoL®), which was firstly developed by Beaudart in 2015 and has been translated into more than 30 languages (i.e., Dutch, Farsi, German, Italian) [124]. The Chinese version of SarQoL® was introduced and validated by Li with excellent consistency (Cronbach’s ∝: 0.867) and re-test reliability (0.997) [125] and will be adopted in this study with the permission of the author.

3.9 Data collection procedure

There are four phases in the procedure: prospective preparation (mainly training for faculty involving in this study), participant recruitment, evaluation (at baseline andpost-intervention), and data storage.

3.9.1 Phase 1: Prospective preparation

In advance of data collection, two group meetings will be held among the PhD student and all other people that will be involved in this study, including research assistants, doctors, and nurses in the CHCCs. In the first group meeting, the general background, objectives, and procedure of this study will be introduced in the meetings. After this, the questionnaires and outcome measurements will be explained in detail to the research assistants. One research assistant will be responsible only for one questionnaire or outcome measurement to avoid the inter-assessor difference. The roleplay and brochures will be used to facilitate their

learning. In the second meeting, the research assistants will practice the questionnaires or outcome measurements until the re-test reliability is more than 90% [126].

3.9.2 Phase 2: Participant recruitment

During the first phase, the inclusion and exclusion criteria have been presented to the research assistants, doctors, and nurses in the CHCCs. Firstly, the doctors and nurses in the CHCCs will find the potential participants and refer them to research assistants, who will explain more about this study and seek initial consent of these older people. After that, the potential participants will be assessed by the research assistants based on the eligible criteria. The eligible participants who are also willing to participate in this study will be required to sign the written consent form.

3.9.3 Phase 3: Evaluation

After consent, the participants will be given an ID number based on the order of consent and assessed for socio-demographic information and outcome measurements. The baseline and post-intervention assessments will be conducted within one week of the recruitment (T0) and cessation of the intervention (T1) separately. All the assessments will be performed by trained research assistants who are blinded to the intervention.

3.9.4 Phase 4: Data storage

Considering the security and confidentiality of the data, the ID number assigned to each participant in Phase 3 which won't contain any personal information will be used. The personal information (i.e., name, address, contacts) will be stored in an encrypted hand disk and only the PhD student and the chief supervisor have the access to it to contact the participants. For the paper questionnaires, they will be lock at the PhD student's office. Besides, considering the willingness of participation of the participants in the control group, all the online sessions will be recorded, and all the materials (brochures, videos) will be provided to them after the intervention. While the recorded videos are mainly

interventionist and researchers appearances, and try not to record the subjects. If we do record the subjects, we will process the videos so that no personal characteristics of the subjects are identified, such as mosaic the subjects' faces or bodies, and process the sound before the videos are provided to participants in the control group.

4.0 Data management

4.0.1 Data entry and screening

The IBM SPSS 23.0 will be used to entry the data to form the database. Before the data entry, a codebook consisting of information of each variable, such as the name of the variable, the meaning of the code, the units of the measurement will be created. All the data will be coded and entered by two research assistants independently, thus forming two databases, which will be checked by a third research assistant. After the data entry, the potential outliers will be detected by using descriptive statistics and Z-score [127]. The maximum and minimum values will be run for categorial data, and the mean and SD will be run for continuous data to initially identify the potential outliers. After that, the Z-score will be utilized to more accurately detect the potential outliners where the Z-score is less than -3 or greater than 3 [128]. If the potential outliers are identified, the manual check between the input-data and the raw data will be conducted.

4.0.2 Missing data

The missing items will be marked as 7777 in SPSS and the number of missing items will be calculated by using the function of SPSS, “Missing value analysis”. Then, little’s Missing Completely at Random (MCAR) test will be used to detect whether the missing value is related to other factors [129].

As the generalized estimating equation (GEE) model will automatically estimate the missing data with all available data and additional imputations will bring bias [130]. the missing date won’t be imputed in this study.

4.0.3 Data analysis

In addition to quantitative data (i.e., questionnaire, physical function tests),

qualitative data (the results of individual interviews to assess the retrospective acceptability and feelings of the participants) will be also contained in this study. The detailed analysis methods are as below:

(1) Quantitative data analysis

The IBM SPSS 23.0 will be used to statistically analyse the data. For continuous variables, the Shapiro-Wilk test will used to evaluate the normality. If the normality is guaranteed, mean and SD will used to describe the data; otherwise, median and interquartile range (IQR: P_{25}, P_{75}) will be used.

For continuous variables in socio-demographic data and baseline outcomes, the independent-samples *t* test will be used if the data normally distribute; otherwise, Mann-Whitney *U* test will be used. Chi square or Fisher test will used for categorical data. For the outcomes, feasibility and acceptability will be presented by rate or absolute number based on the nature of the data, such as the time spent recruiting participants (absolute number), eligibility rate. GEE model will be used to analyse the preliminary effect of this study. Both per-protocol (PP) and intention-to-treat (ITT) analysis will be conducted in this study. As said above, the attention rate >80% is acceptable and will be included in PP analysis.

(2) Qualitative data analysis

The individual interviews will be audio-recorded. The Braun and Clark's thematic approach will be used to analyse the data inductively [131]. The PhD student and an academic researcher who have rich experience in qualitative research will listening to the recordings repeatedly to form the transcript, code it and sort the codes into themes. A final codes framework will be formed by refining and defining the themes repeatedly through group discussion among the two researchers and a third academic researcher. The data will be continuously analysed until there is no theme emerge. The Lincoln and Guba criteria [132] will be followed to improve the rigor of result of qualitative data in this study.

4. Intervention fidelity

Intervention fidelity helps researchers to improve the internal and external

validity and reliability of their study and have greater confidence of the results. The National Institutes of Health (NIH) Behaviour Change Consortium recommended a framework of assessing the intervention fidelity in five aspects, including study design, training providers, delivery of intervention, participants receipt, enactment of treatment skills [79].

4.1 Intervention fidelity for design of study

The intervention fidelity for design of study is to guarantee that a study can test the suggested hypotheses in line with its potential theory. In this study, the HOME program is developed based on our proposed framework and the already tested interventions through literature review, SR, and consultation of experts.

4.2 Intervention fidelity for training provider

The intervention fidelity for training provider is to ensure the providers are well-trained to be capable of delivering the interventions. In this study, one mindfulness instructor with sufficient trainings and qualifications, who are also familiar with physical exercise, will be the intervention providers. Besides, information of this study and the detailed HOME program will be explained to the providers through group meetings and materials of these information will be given to them. Several rehearsals will be performed before the formal intervention.

For RA, they will receive trainings on the content of this study, the use of questionnaires and equipment. The inter/intra-reliability will be assessed by intraclass correlation efficient (ICC) before the start and monthly during the whole project. If ICC is >0.9, the RA will pass the assessment and able to involve in this project.

4.3 Intervention fidelity for delivery

The intervention fidelity for delivery of treatment is to enable the intervention to be delivered as the intended. In this study, all sessions will be video-recorded, and the PhD student will check the consistency of the delivery within one hour after each session. If there is any difference, the providers will be informed to revise it in the next session.

4.4 Intervention fidelity for participants' receipt

The intervention fidelity for participants receipt is to facilitate the participants to understand and perform the interventions. The brochure of detailed intervention will also be provided. During each intervention, the participants will be required to turn on their camera at least 5 minutes to check whether they are doing the correct intervention.

4.5 Intervention fidelity for enactment

The intervention fidelity for enactment skills is to evaluate the ability of participants to perform the intervention related behaviours in real life. In this study, an intervention diary containing the walking and a black page which participants can write down any additional exercise they do in real life will be used.

5. Ethical consideration

The ethical approval will be obtained from the Research Committee of The Hong Kong Polytechnic University before recruiting participants. This study will also be registered at the ClinicalTrials.gov. The principles of the Helsinki Declaration will be followed: autonomy, non-maleficence and beneficence, and confidentiality.

5.1 Autonomy

The information of this study (i.e., objectives, methods, potential benefit, and harm) will be explained to the participants. After learning about this study, they will decide whether to participate in this study or not by their own, and the written informed consent will be obtained if they are willing to join. Besides, they can choose to discontinue this intervention at any time even after the participation. They right to health service in the CHCC won't be influenced.

5.2 Non-maleficence and beneficence

For participants in the control group, they will receive usual care which is harmless. Participants in the intervention group will receive the HOME program. This program is developed by a modification of two already tested interventions which have no adverse even been reported. Besides, the HOME program will also

be reviewed by experts before its use to maximally guarantee its safety. After further testing the feasibility, acceptability and preliminary effect of the HOME program, the participants in the control group will receive all the videos and material of this intervention after the secession of this study.

5.3 Confidentiality

All the collected data (e.g., questionnaire, tests) will be anonymous, and the name of the participants will be replaced by coding. All information collected in this study will be only used for this study and will not be disclosed. When publishing relevant papers, only the research results will be presented, and private information such as the name of the research object will not be disclosed. The data will be kept for three years after the completion of this study and then be destroyed.

6. Significances of this study

This study will adopt the mindfulness-based PE (the HOME program), combining the MBI and the conventional PE, which is novel in the research field related to sarcopenia. The HOME program addresses the limitations of previous studies on sarcopenia by integrating MBI into PE to recognise the key points of human motivation in the PRIME theory of motivation, such as self-awareness, positive feelings and focusing on the present moment. For researchers, this study provides a relatively new sub-area in this field by generating insights on the importance of the above factors and the potential effectiveness of the mindfulness-based PE. For health professionals, the study provides a potentially effective way to improve the motivation and adherence to PE to treat sarcopenia. For older people with sarcopenia, this study has the potential to improve the symptoms of sarcopenia, the motivation and adherence to PE as well as the psychological health of this population, which finally improves the holistic welling of this population.

7. Limitations

This study has several limitations. First, considering practical issues (e.g., time, money), the long-term effects of the mindfulness-based PE intervention won’t be explored. Thus, longer follow-up is needed in further research. Second,

the nature of pilot RCT could undermine the powder of statistical tests for the effectiveness of the HOME program on outcomes. Thus, a full scale RCT is required to test the accurate effectiveness of the HOME program in future. Finally, as this study targets on sarcopenic older people living in the community, the feasibility, acceptability, and effectiveness of the HOME program on sarcopenic older people living in other places (i.e., nursing homes, hospitals) still need to be explored.

8. Summary

This study presents details of a pilot RCT to evaluate the feasibility, acceptability, and preliminary effect of the HOME program in community-dwelling older people with sarcopenia. This will provide the evident for our search objectives to explore the usability of mindfulness-based PE in this population.

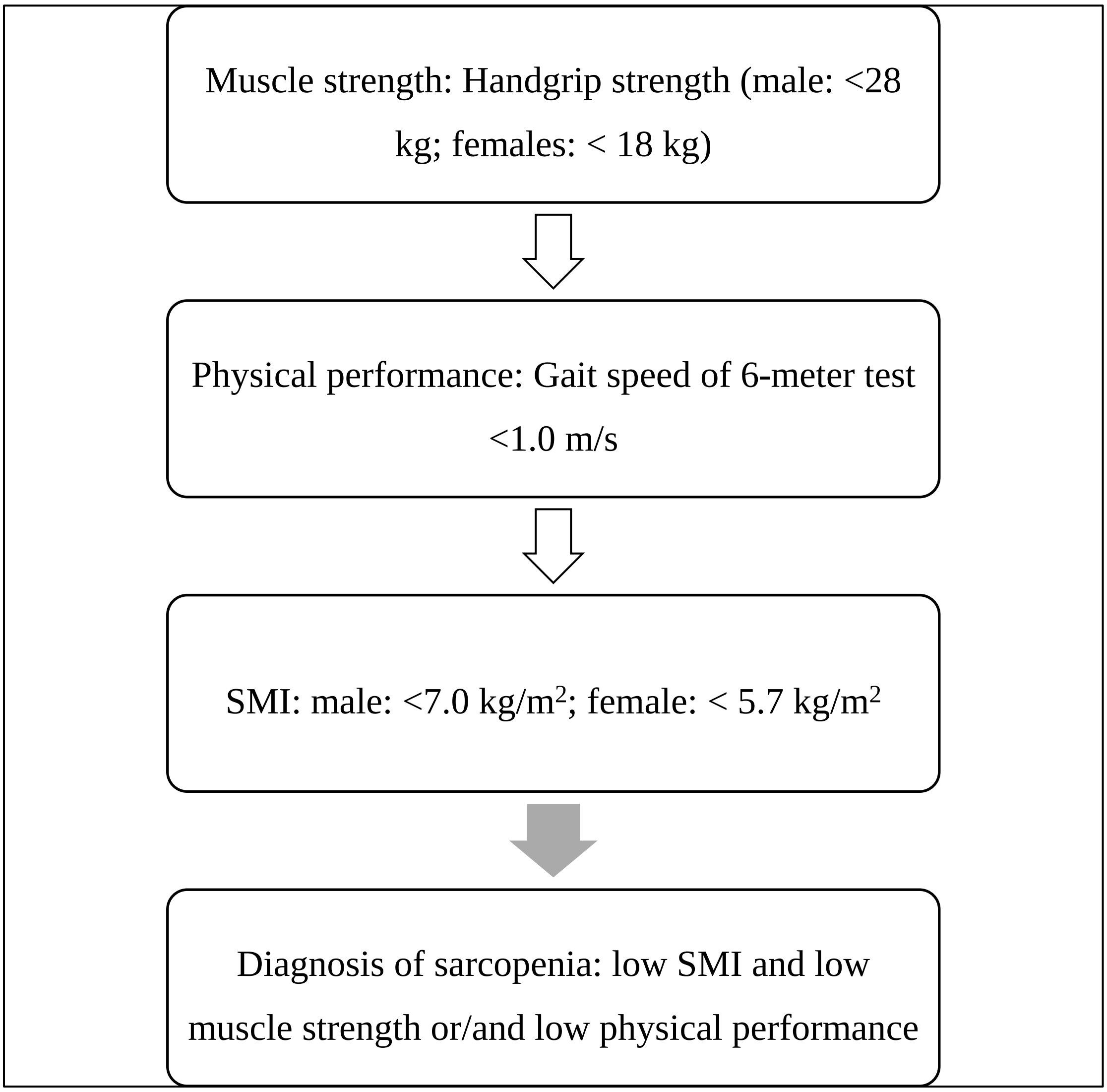

Figure 3 the AWGS 2019 sarcopenia diagnosis procedure

Reference

1. Cruz-Jentoft AJ, Sayer AA: **Sarcopenia**. *Lancet* 2019, **393**(10191):2636-2646.
2. Mayhew AJ, Amog K, Phillips S, Parise G, McNicholas PD, de Souza RJ, Thabane L, Raina P: **The prevalence of sarcopenia in community-dwelling older adults, an exploration of differences between studies and within definitions: a systematic review and meta-analyses**. *Age Ageing* 2019, **48**(1):48-56.
3. Cao L, Morley JE: **Sarcopenia Is Recognized as an Independent Condition by an International Classification of Disease, Tenth Revision, Clinical Modification (ICD-10-CM) Code**. *J Am Med Dir Assoc* 2016, **17**(8):675-677.
4. **Ageing and health** [https://www.who.int/news-room/fact-sheets/detail/ageing-and-health]
5. **Bulletin of the Seventh National Census (No. 5)** [http://www.stats.gov.cn/tjsj/tjgb/rkpcgb/qgrkpcgb/202106/t20210628_1818824.html]
6. Yanan W, Juanlan X, Hongling S: **Influencing factors of sarcopenia among elderly patients**. *Modern Clinical Nursing* 2018, **9**:45-50.
7. Beaudart C, Zaaria M, Pasleau F, Reginster JY, Bruyère O: **Health Outcomes of Sarcopenia: A Systematic Review and Meta-Analysis**. *PLoS One* 2017, **12**(1):e0169548.
8. Peng TC, Chen WL, Wu LW, Chang YW, Kao TW: **Sarcopenia and cognitive impairment: A systematic review and meta-analysis**. *Clin Nutr* 2020, **39**(9):2695-2701.
9. Chang KV, Hsu TH, Wu WT, Huang KC, Han DS: **Is sarcopenia associated with depression? A systematic review and meta-analysis of observational studies**. *Age Ageing* 2017, **46**(5):738-746.
10. Kirkham FA, Bunting E, Fantin F, Zamboni M, Rajkumar C: **Independent Association Between Cardio-Ankle Vascular Index and Sarcopenia in Older U.K. Adults**. *J Am Geriatr Soc* 2019, **67**(2):317-322.
11. Cho HW, Chung W, Moon S, Ryu OH, Kim MK, Kang JG: **Effect of Sarcopenia and Body Shape on Cardiovascular Disease According to Obesity Phenotypes**. *Diabetes Metab J* 2021, **45**(2):209-218.
12. Yang M, Liu Y, Zuo Y, Tang H: **Sarcopenia for predicting falls and hospitalization in community-dwelling older adults: EWGSOP versus EWGSOP2**. *Scientific reports* 2019, **9**(1):17636.
13. Nakamura K, Yoshida D, Honda T, Hata J, Shibata M, Hirakawa Y, Furuta Y, Kishimoto H, Ohara T, Kitazono T *et al*: **Prevalence and Mortality of Sarcopenia in a Community-dwelling Older Japanese Population: The Hisayama Study**. *Journal of epidemiology* 2021, **31**(5):320-327.
14. Su Y, Hirayama K, Han TF, Izutsu M, Yuki M: **Sarcopenia Prevalence and Risk Factors among Japanese Community Dwelling Older Adults Living in a Snow-Covered City According to EWGSOP2**. *J Clin Med* 2019, **8**(3).
15. Tan LF, Lim ZY, Choe R, Seetharaman S, Merchant R: **Screening for Frailty and Sarcopenia Among Older Persons in Medical Outpatient Clinics and its Associations With Healthcare Burden**. *J Am Med Dir Assoc* 2017, **18**(7):583-587.
16. Bruyère O, Beaudart C, Ethgen O, Reginster JY, Locquet M: **The health economics burden of sarcopenia: a systematic review**. *Maturitas* 2019, **119**:61-69.

17. Keller K: **Sarcopenia**. *Wiener Medizinische Wochenschrift* 2019, **169**(7):157-172.
18. Marcos-Pardo PJ, González-Gálvez N, López-Vivancos A, Espeso-García A, Martínez-Aranda LM, Gea-García GM, Orquín-Castrillón FJ, Carbonell-Baeza A, Jiménez-García JD, Velázquez-Díaz D *et al*: **Sarcopenia, Diet, Physical Activity and Obesity in European Middle-Aged and Older Adults: The LifeAge Study**. *Nutrients* 2021, **13**(1).
19. Chen L-K, Woo J, Assantachai P, Auyeung T-W, Chou M-Y, Iijima K, Jang HC, Kang L, Kim M, Kim S *et al*: **Asian Working Group for Sarcopenia: 2019 Consensus Update on Sarcopenia Diagnosis and Treatment**. *J Am Med Dir Assoc* 2020, **21**(3).
20. Di Girolamo FG, Fiotti N, Milanović Z, Situlin R, Mearelli F, Vinci P, Šimunič B, Pišot R, Narici M, Biolo G: **The Aging Muscle in Experimental Bed Rest: A Systematic Review and Meta-Analysis**. *Front Nutr* 2021, **8**:633987.
21. Hurst C, Robinson SM, Witham MD, Dodds RM, Granic A, Buckland C, De Biase S, Finnegan S, Rochester L, Skelton DA: **Resistance exercise as a treatment for sarcopenia: prescription and delivery**. *Age and ageing* 2022, **51**(2):afac003.
22. Wu PY, Huang KS, Chen KM, Chou CP, Tu YK: **Exercise, Nutrition, and Combined Exercise and Nutrition in Older Adults with Sarcopenia: A Systematic Review and Network Meta-analysis**. *Maturitas* 2021, **145**:38-48.
23. Bao W, Sun Y, Zhang T, Zou L, Wu X, Wang D, Chen Z: **Exercise Programs for Muscle Mass, Muscle Strength and Physical Performance in Older Adults with Sarcopenia: A Systematic Review and Meta-Analysis**. *Aging Dis* 2020, **11**(4):863-873.
24. de Mello RGB, Dalla Corte RR, Gioscia J, Moriguchi EH: **Effects of Physical Exercise Programs on Sarcopenia Management, Dynapenia, and Physical Performance in the Elderly: A Systematic Review of Randomized Clinical Trials**. *J Aging Res* 2019, **2019**:1959486.
25. Lozano-Montoya I, Correa-Perez A, Abraha I, Soiza RL, Cherubini A, O'Mahony D, Cruz-Jentoft AJ: **Nonpharmacological interventions to treat physical frailty and sarcopenia in older patients: a systematic overview - the SENATOR Project ONTOP Series**. *Clinical Interventions in Aging* 2017, **12**:721-740.
26. Yoshimura Y, Wakabayashi H, Yamada M, Kim H, Harada A, Arai H: **Interventions for Treating Sarcopenia: A Systematic Review and Meta-Analysis of Randomized Controlled Studies**. *J Am Med Dir Assoc* 2017, **18**(6):553.e551-553.e516.
27. Lin T-R, Huang X-Y, Hwu C-M: **Exercise Experiences of Older Adults with Diabetes and Sarcopenia: A Phenomenological Study**. *Clinical Nursing Research* 2021:10547738211039381.
28. Dorresteijn TA, Rixt Zijlstra GA, Van Eijs YJ, Vlaeyen JW, Kempen GI: **Older people's preferences regarding programme formats for managing concerns about falls**. *Age and ageing* 2012, **41**(4):474-481.
29. Sen EI, Eyigor S, Dikici Yagli M, Ozcete ZA, Aydin T, Kesiktas FN, Aydin FY, Vural M, Sahin N, Karan A: **Effect of Home-Based Exercise Program on Physical Function and Balance in Older Adults With Sarcopenia: A Multicenter Randomized Controlled Study**. *J Aging Phys Act* 2021, **29**(6):1010-1017.
30. Farrance C, Tsofliou F, Clark C: **Adherence to community based group exercise interventions for older people: A mixed-methods systematic review**. *Preventive Medicine* 2016, **87**:155-166.

31. Essery R, Geraghty AW, Kirby S, Yardley L: **Predictors of adherence to home-based physical therapies: a systematic review**. *Disabil Rehabil* 2017, **39**(6):519-534.
32. Papadopoulou SK, Tsintavis P, Potsaki P, Papandreou D: **Differences in the Prevalence of Sarcopenia in Community-Dwelling, Nursing Home and Hospitalized Individuals. A Systematic Review and Meta-Analysis**. *J Nutr Health Aging* 2020, **24**(1):83-90.
33. Herrema AL, Westerman MJ, van Dongen EJ, Kudla U, Veltkamp M: **Combined protein-rich diet with resistance exercise intervention to counteract sarcopenia: A qualitative study on drivers and barriers of compliance**. *Journal of aging and physical activity* 2018, **26**(1):106-113.
34. Thiebaud RS, Funk MD, Abe T: **Home-based resistance training for older adults: A systematic review**. *Geriatrics & Gerontology International* 2014, **14**(4):750-757.
35. Mañas A, Gómez-Redondo P, Valenzuela PL, Morales JS, Lucía A, Ara I: **Unsupervised home-based resistance training for community-dwelling older adults: A systematic review and meta-analysis of randomized controlled trials**. *Ageing Res Rev* 2021, **69**:101368.
36. Izquierdo M, Merchant R, Morley J, Anker S, Aprahamian I, Arai H, Aubertin-Leheudre M, Bernabei R, Cadore E, Cesari M: **International exercise recommendations in older adults (ICFSR): expert consensus guidelines**. *The journal of nutrition, health & aging* 2021, **25**(7):824-853.
37. West R: **Towards a comprehensive theory of addiction**. *Drugs and Alcohol Today* 2006, **6**(1):28-32.
38. Chapman P, van Loon E, Trawley S, Crundall D: **A comparison of drivers' eye movements in filmed and simulated dangerous driving situations**. In: *Behavioural research in road safety, 2007: Seventeenth seminar: 2007*; 2007: 112-123.
39. Dismore L, Hurst C, Sayer AA, Stevenson E, Aspray T, Granic A: **Study of the older adults' motivators and barriers engaging in a nutrition and resistance exercise intervention for sarcopenia: an embedded qualitative project in the MIlkMAN Pilot study**. *Gerontology and Geriatric Medicine* 2020, **6**:2333721420920398.
40. Kwon YN, Yoon SS: **Sarcopenia: Neurological Point of View**. *jbm* 2017, **24**(2):83-89.
41. Billot M, Calvani R, Urtamo A, Sánchez-Sánchez JL, Ciccolari-Micaldi C, Chang M, Roller-Wirnsberger R, Wirnsberger G, Sinclair A, Vaquero-Pinto N *et al*: **Preserving Mobility in Older Adults with Physical Frailty and Sarcopenia: Opportunities, Challenges, and Recommendations for Physical Activity Interventions**. *Clinical interventions in aging* 2020, **15**:1675-1690.
42. Ahmad MH, Shahar S, Teng NIMF, Manaf ZA, Sakian NIM, Omar B: **Applying theory of planned behavior to predict exercise maintenance in sarcopenic elderly**. *Clinical interventions in aging* 2014, **9**:1551.
43. Nilsson MI, Mikhail A, Lan L, Di Carlo A, Hamilton B, Barnard K, Hettinga BP, Hatcher E, Tarnopolsky MG, Nederveen JP *et al*: **A Five-Ingredient Nutritional Supplement and Home-Based Resistance Exercise Improve Lean Mass and Strength in Free-Living Elderly**. *Nutrients* 2020, **12**(8).
44. Abdelbasset WK: **Stay Home: Role of Physical Exercise Training in Elderly Individuals' Ability to Face the COVID-19 Infection**. *J Immunol Res* 2020, **2020**:8375096.
45. Ammar A, Brach M, Trabelsi K, Chtourou H, Boukhris O, Masmoudi L, Bouaziz B, Bentlage

E, How D, Ahmed M *et al*: **Effects of COVID-19 Home Confinement on Eating Behaviour and Physical Activity: Results of the ECLB-COVID19 International Online Survey**. *Nutrients* 2020, **12**(6).

46. Schuch FB, Bulzing RA, Meyer J, López-Sánchez GF, Grabovac I, Willeit P, Vancampfort D, Caperchione CM, Sadarangani KP, Werneck AO *et al*: **Moderate to vigorous physical activity and sedentary behavior changes in self-isolating adults during the COVID-19 pandemic in Brazil: a cross-sectional survey exploring correlates**. *Sport Sci Health* 2021:1-9.
47. West R: **The multiple facets of cigarette addiction and what they mean for encouraging and helping smokers to stop**. *COPD: Journal of Chronic Obstructive Pulmonary Disease* 2009, **6**(4):277-283.
48. West R, Michie S: **A brief introduction to the COM-B Model of behaviour and the PRIME Theory of motivation**. *Qeios* 2020.
49. Ullmann G, Li Y, Ray MA, Lee ST: **Study protocol of a randomized intervention study to explore effects of a pure physical training and a mind–body exercise on cognitive executive function in independent living adults age 65–85**. *Aging Clinical and Experimental Research* 2021, **33**(5):1259-1266.
50. Hassan BH, Hewitt J, Keogh JWL, Bermeo S, Duque G, Henwood TR: **Impact of resistance training on sarcopenia in nursing care facilities: A pilot study**. *Geriatric Nursing* 2016, **37**(2):116-121.
51. Reangsing C, Rittiwong T, Schneider JK: **Effects of mindfulness meditation interventions on depression in older adults: A meta-analysis**. *Aging & Mental Health* 2021, **25**(7):1181-1190.
52. Li Z, Tong X, Ma Y, Bao T, Yue J: **Prevalence of depression in patients with sarcopenia and correlation between the two diseases: systematic review and meta-analysis**. *Journal of cachexia, sarcopenia and muscle* 2022.
53. Moorey S: **The six cycles maintenance model: growing a "vicious flower" for depression**. *Behavioural and cognitive psychotherapy* 2010, **38**(2):173-184.
54. de Zwart PL, Jeronimus BF, de Jonge P: **Empirical evidence for definitions of episode, remission, recovery, relapse and recurrence in depression: a systematic review**. *Epidemiol Psychiatr Sci* 2019, **28**(5):544-562.
55. Ohrnberger J, Fichera E, Sutton M: **The dynamics of physical and mental health in the older population**. *The Journal of the Economics of Ageing* 2017, **9**:52-62.
56. Zimmermann FF, Burrell B, Jordan J: **The acceptability and potential benefits of mindfulness-based interventions in improving psychological well-being for adults with advanced cancer: A systematic review**. *Complementary therapies in clinical practice* 2018, **30**:68-78.
57. Goldberg SB, Tucker RP, Greene PA, Davidson RJ, Wampold BE, Kearney DJ, Simpson TL: **Mindfulness-based interventions for psychiatric disorders: A systematic review and meta-analysis**. *Clin Psychol Rev* 2018, **59**:52-60.
58. Morone NE, Greco CM: **Mind–Body Interventions for Chronic Pain in Older Adults: A Structured Review**. *Pain Medicine* 2007, **8**(4):359-375.
59. Rusch HL, Rosario M, Levison LM, Olivera A, Livingston WS, Wu T, Gill JM: **The effect of mindfulness meditation on sleep quality: a systematic review and meta-analysis of**

randomized controlled trials. *Ann N Y Acad Sci* 2019, **1445**(1):5-16.
60. Wang S, Yin H, Jia Y, Zhao L, Wang L, Chen L: **Effects of Mind-Body Exercise on Cognitive Function in Older Adults With Cognitive Impairment: A Systematic Review and Meta-analysis**. *The Journal of Nervous and Mental Disease* 2018, **206**(12):913-924.
61. Kwok JY, Kwan JC, Auyeung M, Mok VC, Lau CK, Choi K, Chan HY: **Effects of mindfulness yoga vs stretching and resistance training exercises on anxiety and depression for people with Parkinson disease: a randomized clinical trial**. *JAMA neurology* 2019, **76**(7):755-763.
62. Ruffault A, Bernier M, Juge N, Fournier JF: **Mindfulness May Moderate the Relationship Between Intrinsic Motivation and Physical Activity: A Cross-Sectional Study**. *Mindfulness* 2016, **7**(2):445-452.
63. Hardison ME, Roll SC: **Mindfulness Interventions in Physical Rehabilitation: A Scoping Review**. *American Journal of Occupational Therapy* 2016, **70**(3).
64. Tsafou KE, Lacroix JP, van Ee R, Vinkers CD, De Ridder DT: **The relation of trait and state mindfulness with satisfaction and physical activity: A cross-sectional study in 305 Dutch participants**. *J Health Psychol* 2017, **22**(10):1221-1232.
65. Schneider J, Malinowski P, Watson PM, Lattimore P: **The role of mindfulness in physical activity: a systematic review**. *Obesity Reviews* 2019, **20**(3):448-463.
66. Yang CH, Conroy DE: **Mindfulness and physical activity: a systematic review and hierarchical model of mindfulness**. *International Journal of Sport and Exercise Psychology* 2020, **18**(6):794-817.
67. Robin N, Toussaint L, Sinnapah S, Hue O, Coudevylle GR: **Beneficial Influence of Mindfulness Training Promoted by Text Messages on Self-Reported Aerobic Physical Activity in Older Adults: A Randomized Controlled Study**. *Journal of Aging and Physical Activity* 2020, **28**(3):406-414.
68. Yang CH, Conroy DE: **Feasibility of an Outdoor Mindful Walking Program for Reducing Negative Affect in Older Adults**. *J Aging Phys Act* 2018:1-10.
69. Roychowdhury D: **Moving Mindfully: The Role of Mindfulness Practice in Physical Activity and Health Behaviours**. *Journal of Functional Morphology and Kinesiology* 2021, **6**(1):19.
70. Bixby WR, Lochbaum MR: **Affect responses to acute bouts of aerobic exercise in fit and unfit participants: An examination of opponent-process theory**. *Journal of Sport Behavior* 2006, **29**(2):111.
71. Roberts KC, Danoff-Burg S: **Mindfulness and Health Behaviors: Is Paying Attention Good for You?** *Journal of American College Health* 2010, **59**(3):165-173.
72. Ye Q, Lin T, Yang Y: **Research progress of health education methods for preventing the elderly from falling at home** *Chinese Nursing Research* 2021, **35**:1944-1946.
73. Yang X, Kovarik CL: **A systematic review of mobile health interventions in China: Identifying gaps in care**. *J Telemed Telecare* 2021, **27**(1):3-22.
74. Man Z, Yue L: **Research on the Value Confirmation and Advancing Strategies for Health Education of the Rural Elderly in the New Era**. *Continue Education Research* 2022, **4**:29-35.
75. Langeard A, Bigot L, Maffiuletti NA, Moussay S, Sesboüé B, Quarck G, Gauthier A: **Non-inferiority of a home-based videoconference physical training program in**

comparison with the same program administered face-to-face in healthy older adults: the MOTION randomised controlled trial. *Age Ageing* 2022, **51**(3).

76. McGuire R, Duncan K, Pozehl B: **Incorporating Intervention Fidelity Components into RCT Promoting Exercise Adherence in Heart Failure Patients**. *Research in nursing & health* 2019, **42**(4):306.
77. Tsekoura M, Billis E, Tsepis E, Dimitriadis Z, Matzaroglou C, Tyllianakis M, Panagiotopoulos E, Gliatis J: **The Effects of Group and Home-Based Exercise Programs in Elderly with Sarcopenia: A Randomized Controlled Trial**. *J Clin Med* 2018, **7**(12).
78. Wang PY, Li Y, Wang Q: **Sarcopenia: An underlying treatment target during the COVID-19 pandemic**. *Nutrition* 2021, **84**:111104.
79. Bellg AJ, Borrelli B, Resnick B, Hecht J, Minicucci DS, Ory M, Ogedegbe G, Orwig D, Ernst D, Czajkowski S: **Enhancing treatment fidelity in health behavior change studies: best practices and recommendations from the NIH Behavior Change Consortium**. *Health psychology* 2004, **23**(5):443.
80. Zhang Y, Liu F, Han J, Wang L, LI X: **Application and research progress of mobile medicine in elderly chronic disease management**. *Chinese Clinical Nursing* 2021, **13**:454-460.
81. Chang KV, Wu WT, Huang KC, Han DS: **Effectiveness of early versus delayed exercise and nutritional intervention on segmental body composition of sarcopenic elders - A randomized controlled trial**. *Clin Nutr* 2021, **40**(3):1052-1059.
82. Li Z, Cui M, Yu K, Zhang X, Li C, Nie X, Wang F: **Effects of nutrition supplementation and physical exercise on muscle mass, muscle strength and fat mass among sarcopenic elderly: a randomized controlled trial**. *Applied physiology, nutrition & metabolism* 2021, **46**(5):494-500.
83. Buyl R, Beogo I, Fobelets M, Deletroz C, Van Landuyt P, Dequanter S, Gorus E, Bourbonnais A, Giguère A, Lechasseur K *et al*: **e-Health interventions for healthy aging: a systematic review**. *Syst Rev* 2020, **9**(1):128.
84. Valenzuela T, Okubo Y, Woodbury A, Lord SR, Delbaere K: **Adherence to Technology-Based Exercise Programs in Older Adults: A Systematic Review**. *J Geriatr Phys Ther* 2018, **41**(1):49-61.
85. van den Helder J, Mehra S, van Dronkelaar C, Ter Riet G, Tieland M, Visser B, Kröse BJA, Engelbert RHH, Weijs PJM: **Blended home-based exercise and dietary protein in community-dwelling older adults: a cluster randomized controlled trial**. *J Cachexia Sarcopenia Muscle* 2020, **11**(6):1590-1602.
86. Luan H, Wang M, Sokol RL, Wu S, Victor BG, Perron BE: **A scoping review of WeChat to facilitate professional healthcare education in Mainland China**. *Med Educ Online* 2020, **25**(1):1782594.
87. **WeChat Data Report (2018)** [https://support.weixin.qq.com/cgi-bin/mmsupport-bin/getopendays]
88. Jiang Y, Liu F, Guo J, Sun P, Chen Z, Li J, Cai L, Zhao H, Gao P, Ding Z *et al*: **Evaluating an Intervention Program Using WeChat for Patients With Chronic Obstructive Pulmonary Disease: Randomized Controlled Trial**. *J Med Internet Res* 2020, **22**(4):e17089.
89. Feng S, Liang Z, Zhang R, Liao W, Chen Y, Fan Y, Li H: **Effects of mobile phone WeChat**

services improve adherence to corticosteroid nasal spray treatment for chronic rhinosinusitis after functional endoscopic sinus surgery: a 3-month follow-up study. *European Archives of Oto-Rhino-Laryngology* 2017, **274**(3):1477-1485.
90. Chen X, Zhou X, Li H, Li J, Jiang H: **The value of WeChat application in chronic diseases management in China**. *Comput Methods Programs Biomed* 2020, **196**:105710.
91. Liu G, Wang S, Liao J, Ou P, Huang L, Xie N, He Y, Lin J, He HG, Hu R: **The Efficacy of WeChat-Based Parenting Training on the Psychological Well-being of Mothers With Children With Autism During the COVID-19 Pandemic: Quasi-Experimental Study**. *JMIR Ment Health* 2021, **8**(2):e23917.
92. Guo Y, Hong YA, Cai W, Li L, Hao Y, Qiao J, Xu Z, Zhang H, Zeng C, Liu C *et al*: **Effect of a WeChat-Based Intervention (Run4Love) on Depressive Symptoms Among People Living With HIV in China: A Randomized Controlled Trial**. *J Med Internet Res* 2020, **22**(2):e16715.
93. Chen J, Ho E, Jiang Y, Whittaker R, Yang T, Bullen C: **Mobile Social Network-Based Smoking Cessation Intervention for Chinese Male Smokers: Pilot Randomized Controlled Trial**. *JMIR Mhealth Uhealth* 2020, **8**(10):e17522.
94. Li X, Li T, Chen J, Xie Y, An X, Lv Y, Lin A: **A WeChat-Based Self-Management Intervention for Community Middle-Aged and Elderly Adults with Hypertension in Guangzhou, China: A Cluster-Randomized Controlled Trial**. *Int J Environ Res Public Health* 2019, **16**(21).
95. Bi J, Yang W, Hao P, Zhao Y, Wei D, Sun Y, Lin Y, Sun M, Chen X, Luo X *et al*: **WeChat as a Platform for Baduanjin Intervention in Patients With Stable Chronic Obstructive Pulmonary Disease in China: Retrospective Randomized Controlled Trial**. *JMIR Mhealth Uhealth* 2021, **9**(2):e23548.
96. Skivington K, Matthews L, Simpson SA, Craig P, Baird J, Blazeby JM, Boyd KA, Craig N, French DP, McIntosh E *et al*: **A new framework for developing and evaluating complex interventions: update of Medical Research Council guidance**. *BMJ* 2021, **374**:n2061.
97. Chodzko-Zajko W, Medicine ACoS: **ACSM's exercise for older adults**: Lippincott Williams & Wilkins; 2013.
98. **Physical activity** [https://www.who.int/news-room/fact-sheets/detail/physical-activity]
99. Greenberg J, Popok PJ, Lin A, Kulich RJ, James P, Macklin EA, Millstein RA, Edwards RR, Vranceanu A-M: **A Mind-Body Physical Activity Program for Chronic Pain With or Without a Digital Monitoring Device: Proof-of-Concept Feasibility Randomized Controlled Trial**. *JMIR Form Res* 2020, **4**(6):e18703.
100. Lam SC, Wong YY, Woo J: **Reliability and validity of the abbreviated mental test (Hong Kong version) in residential care homes**. *J Am Geriatr Soc* 2010, **58**(11):2255-2257.
101. Whitehead AL, Julious SA, Cooper CL, Campbell MJ: **Estimating the sample size for a pilot randomised trial to minimise the overall trial sample size for the external pilot and main trial for a continuous outcome variable**. *Statistical methods in medical research* 2016, **25**(3):1057-1073.
102. Cramer JT, Cruz-Jentoft AJ, Landi F, Hickson M, Zamboni M, Pereira SL, Hustead DS, Mustad VA: **Impacts of High-Protein Oral Nutritional Supplements Among Malnourished Men and Women with Sarcopenia: A Multicenter, Randomized, Double-Blinded, Controlled Trial**. *J Am Med Dir Assoc* 2016, **17**(11):1044-1055.

103. Hertzog MA: **Considerations in determining sample size for pilot studies**. *Research In Nursing & Health* 2008, **31**(2):180-191.
104. Sekhon M, Cartwright M, Francis JJ: **Acceptability of healthcare interventions: an overview of reviews and development of a theoretical framework**. *BMC Health Services Research* 2017, **17**(1):88.
105. Ortlipp M: **Keeping and using reflective journals in the qualitative research process**. *The qualitative report* 2008, **13**(4):695-705.
106. Fess EE: **American society of hand therapists**. *The Journal of Hand Surgery* 1983, **8**(5):625-627.
107. Liu JD, Chung P-K, Zhang C-Q, Si G: **Chinese-translated Behavioral Regulation in Exercise Questionnaire-2: evidence from university students in the Mainland and Hong Kong of China**. *Journal of Sport and Health Science* 2015, **4**(3):228-234.
108. Herrmann N, Mittmann N, Silver IL, Shulman KI, Busto UA, Shear NH, Naranjo CA: **A validation study of the Geriatric Depression Scale short form**. *International Journal of Geriatric Psychiatry* 1996, **11**(5):457-460.
109. Yesavage JA, Brink TL, Rose TL, Lum O, Huang V, Adey M, Leirer VO: **Development and validation of a geriatric depression screening scale: a preliminary report**. *Journal of psychiatric research* 1982, **17**(1):37-49.
110. Nyunt MSZ, Fones C, Niti M, Ng T-P: **Criterion-based validity and reliability of the Geriatric Depression Screening Scale (GDS-15) in a large validation sample of community-living Asian older adults**. *Aging and Mental Health* 2009, **13**(3):376-382.
111. Sugishita K, Sugishita M, Hemmi I, Asada T, Tanigawa T: **A validity and reliability study of the Japanese version of the Geriatric Depression Scale 15 (GDS-15-J)**. *Clinical Gerontologist* 2017, **40**(4):233-240.
112. Dias FLdC, Teixeira AL, Guimarães HC, Barbosa MT, Resende EdPF, Beato RG, Carmona KC, Caramelli P: **Accuracy of the 15-item Geriatric Depression Scale (GDS-15) in a community-dwelling oldest-old sample: the Pietà Study**. *Trends in psychiatry and psychotherapy* 2017, **39**:276-279.
113. Dan T: **Application of Short Form Geriatric Depression Scale (GDS-15) In Chinese Elderly**. *Application of Short Form Geriatric Depression Scale (GDS-15) In Chinese Elderly* 2013, **21**:402-405.
114. LIU S: **The relationship between frailty and depression in elderly hospitalized patients**. Peking Union Medical College; 2019.
115. TAN S: **The longitudinal relationship between test stress and psychological well-being in adolescents: the mediating role of self-esteem**. Shaanxi Normal University; 2016.
116. Cheng S-T, Chan AC: **Measuring psychological well-being in the Chinese**. *Personality and Individual Differences* 2005, **38**(6):1307-1316.
117. Washburn RA, Smith KW, Jette AM, Janney CA: **The Physical Activity Scale for the Elderly (PASE): development and evaluation**. *J Clin Epidemiol* 1993, **46**(2):153-162.
118. Ngai SP, Cheung RT, Lam PL, Chiu JK, Fung EY: **Validation and reliability of the Physical Activity Scale for the Elderly in Chinese population**. *J Rehabil Med* 2012, **44**(5):462-465.
119. Ku P-W, Sun W-J, Chang C-Y, Chen L-J: **Reliability and validity of the Chinese version of the physical activity scale for the elderly**. *大專體育學刊* 2013, **15**(3):309-319.

120. Baer RA, Smith GT, Hopkins J, Krietemeyer J, Toney L: **Using self-report assessment methods to explore facets of mindfulness**. *Assessment* 2006, **13**(1):27-45.
121. Deng Y-Q, Liu X-H, Rodriguez MA, Xia C-Y: **The Five Facet Mindfulness Questionnaire: Psychometric Properties of the Chinese Version**. *Mindfulness* 2011, **2**(2):123-128.
122. Hou J, Wong SY-S, Lo HH-M, Mak WW-S, Ma HS-W: **Validation of a Chinese version of the Five Facet Mindfulness Questionnaire in Hong Kong and development of a short form**. *Assessment* 2014, **21**(3):363-371.
123. Zhu T, Chen C, Chen S: **Validation of a Chinese version of the five facet mindfulness questionnaire and development of a short form based on item response theory**. *Current Psychology* 2021:1-13.
124. **Sarcopenia and Quality of life** [https://sarqol.org/en/sarqol_form]
125. Le X, Wei Y, Hao D, Shan L, Li X, Shi Q, Ding D, Cheng X, Lim HLE, Ng BY: **Psychometric properties of the Chinese version of the sarcopenia and quality of life, a quality of life questionnaire specific for sarcopenia**. *Calcified Tissue International* 2021, **109**(4):415-422.
126. Polit DF, Beck CT, Owen SV: **Is the CVI an acceptable indicator of content validity? Appraisal and recommendations**. *Research in nursing & health* 2007, **30**(4):459-467.
127. Shiffler RE: **Maximum Z scores and outliers**. *The American Statistician* 1988, **42**(1):79-80.
128. Williams P: **Interactive statistics**. *Sunderland, MA: Sinauer Associates* 2004.
129. Little RJ: **A test of missing completely at random for multivariate data with missing values**. *Journal of the American statistical Association* 1988, **83**(404):1198-1202.
130. Little RJ, Rubin DB: **Statistical analysis with missing data**, vol. 793: John Wiley & Sons; 2019.
131. Braun V, Clarke V: **One size fits all? What counts as quality practice in (reflexive) thematic analysis?** *Qualitative research in psychology* 2021, **18**(3):328-352.
132. Schwandt TA, Lincoln YS, Guba EG: **Judging interpretations: But is it rigorous? Trustworthiness and authenticity in naturalistic evaluation**. *New directions for evaluation* 2007, **114**:11-25.
