# Supplementary figures and images for: Feasibility and preliminary effects of a mindfulness-based physical exercise (MBPE) program for community-dwelling older people with sarcopenia: A protocol for a parallel, two-armed pilot randomised controlled trial
Source: PLoS One. 2024 Apr 18;19(4):e0302235. doi: 10.1371/journal.pone.0302235 (PMC11025830; doi:10.1371/journal.pone.0302235)

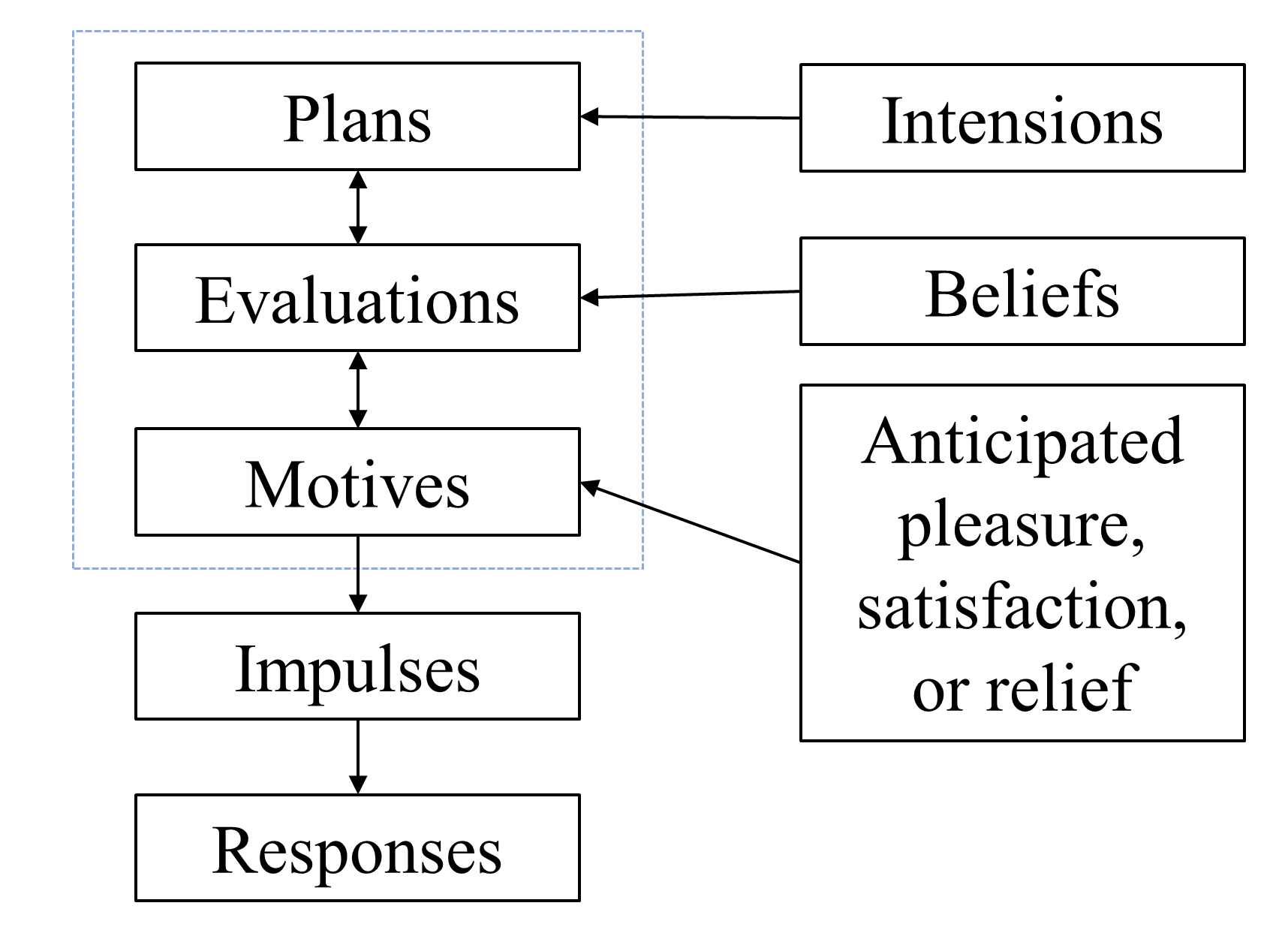

Supplement: S1 Fig — (TIF) [file pone.0302235.s004.tif]

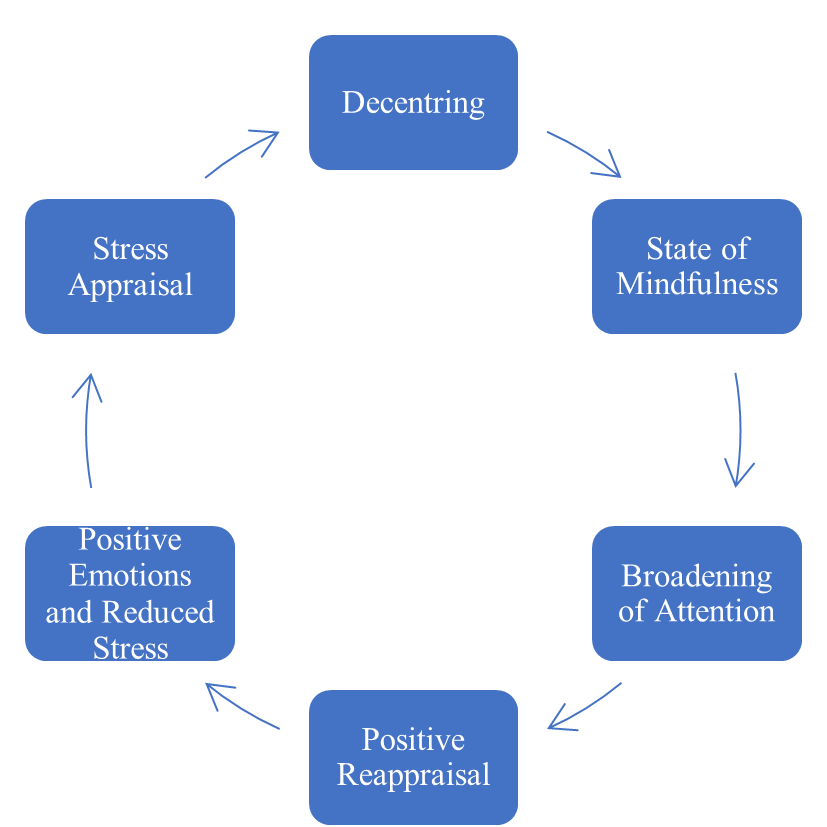

Supplement: S2 Fig — (TIF) [file pone.0302235.s005.tif]
